# Evaluation of linear and non-linear activation dynamics models for insect muscle

**DOI:** 10.1371/journal.pcbi.1007437

**Published:** 2019-10-14

**Authors:** Nalin Harischandra, Anthony J. Clare, Jure Zakotnik, Laura M. L. Blackburn, Tom Matheson, Volker Dürr

**Affiliations:** 1 Biological Cybernetics, Faculty of Biology, Bielefeld University, Bielefeld, Germany; 2 Cognitive Interaction Technology—Center of Excellence (CITEC), Bielefeld University, Bielefeld, Germany; 3 University of Leicester, Department of Neuroscience, Psychology and Behaviour, Leicester, United Kingdom; 4 Department of Zoology, University of Cambridge, Cambridge, United Kingdom; Northeastern University, UNITED STATES

## Abstract

In computational modelling of sensory-motor control, the dynamics of muscle contraction is an important determinant of movement timing and joint stiffness. This is particularly so in animals with many slow muscles, as is the case in insects—many of which are important models for sensory-motor control. A muscle model is generally used to transform motoneuronal input into muscle force. Although standard models exist for vertebrate muscle innervated by many motoneurons, there is no agreement on a parametric model for single motoneuron stimulation of invertebrate muscle. Although several different models have been proposed, they have never been evaluated using a common experimental data set. We evaluate five models for isometric force production of a well-studied model system: the locust hind leg tibial extensor muscle. The response of this muscle to motoneuron spikes is best modelled as a non-linear low-pass system. Linear first-order models can approximate isometric force time courses well at high spike rates, but they cannot account for appropriate force time courses at low spike rates. A linear third-order model performs better, but only non-linear models can account for frequency-dependent change of decay time and force potentiation at intermediate stimulus frequencies. Some of the differences among published models are due to differences among experimental data sets. We developed a comprehensive toolbox for modelling muscle activation dynamics, and optimised model parameters using one data set. The “Hatze-Zakotnik model” that emphasizes an accurate single-twitch time course and uses frequency-dependent modulation of the twitch for force potentiation performs best for the slow motoneuron. Frequency-dependent modulation of a single twitch works less well for the fast motoneuron. The non-linear “Wilson” model that optimises parameters to all data set parts simultaneously performs better here. Our open-access toolbox provides powerful tools for researchers to fit appropriate models to a range of insect muscles.

## Author summary

Insects are important study organisms in the neuroscience of sensory-motor systems. Since the dynamics of muscle contraction and associated changes in force, torque or stiffness are central to our understanding of sensory-motor systems in general, the choice of the most appropriate model for insect muscle matters. Computational modelling of muscle properties typically separates activation dynamics from contraction dynamics. The former models the development of muscle force in response to motoneuron activity, whereas the latter describes how this force is affected by the current physical state of the muscle: its length and contraction velocity. We evaluate five published activation dynamics models for insect muscle. We explain differences between them, suggest how to decide which one to use, and provide an open-source toolbox for activation dynamics modelling. We further show that non-linear models are the best choice if: (i) the time course of a single muscle twitch is slow, (ii) the spike frequency ranges between one and thirty spikes per second, or (iii) the sensory-motor system tends to execute movements in a similar manner even if the demand on joint torque or stiffness changes.

## Introduction

The small number of motoneurons per muscle in insects has permitted detailed analyses of context-dependent adaptation of motor patterns and behaviour in several model species. Counter-intuitively, however, this small number of motoneurons poses a problem for the modelling of sensory-motor control, and the computational neuroscience of behaviour in general. This is because it necessitates accurate modelling of the spike-by-spike activation dynamics of the muscle, rather than simpler modelling based on average motor spike rate. Several muscle activation models have been proposed for insect muscle [1–4], but these have very different properties. Furthermore, although their published parameters were obtained from fits to experimental data on the same leg muscle (extensor tibiae) and, in three of four cases, the same leg and species (the metathoracic jumping leg of the locust *Schistocerca gregaria* [1, 3, 4]), the models yield very different force time courses when driven with identical inputs. This calls for a comparative evaluation. To identify the most reliable and computationally sound model of insect muscle activation dynamics, we provide a comprehensive muscle activation model toolbox for Matlab, comprising three linear and two non-linear models. We use this toolbox to identify differences caused by the models themselves, as opposed to effects caused by differences in the sample data used to tune the published versions of the models. To this end, we use a data set [5] comprising isometric force contraction time courses in response to metathoracic fast extensor tibiae (FETi) and slow extensor tibiae (SETi) motoneuron stimulation in the locust. In particular, we compare the model predictions of frequency-dependencies of maximum force, rise- and decay times using our own and published experimental data. Understanding arthropod muscle and being able to model its actions is important, because insects, crustaceans and spiders are widely and increasingly being used as models for biomimetic and robotic applications (e.g., flight: [6]; walking: [7, 8]; jumping: [9]). The active and passive properties of arthropod muscle provide remarkable solutions to the conflicting demands of flexibility and stability of movement, driven by relatively simple nervous control systems. Engineers struggle to manufacture equally versatile actuators, in part because the properties of muscles and their patterns of activation and modulation are still not fully understood.

Muscle models come in two types: ‘physiological’ and ‘conceptual’. Physiological models describe the molecular mechanisms underlying muscle contraction, e.g., by formal description of calcium release dynamics, binding-unbinding kinetics and diffusion processes. Such models can predict muscle forces accurately and reproduce effects such as potentiation and depression [10,11], but often contain many parameters that are unknown or difficult to determine (indirectly) from force measurements or behavioural data. Conceptual models, on the other hand, estimate the transfer function from motoneuron spike activity (input) to muscle contraction (output). Generally, they have fewer parameters because they do not model details of the underlying physiological mechanisms. In some cases, they may not model any physiological mechanism at all but instead implement an abstract transfer function [12].

The generation of muscle force is typically modelled as a two-stage process that separates the *activation dynamics* of muscle from its *contraction dynamics* [13]. *Contraction dynamics* describe how much force is produced given the active state and the current muscle length and any length change, taking into account the force-length-velocity characteristics of the muscle, and the elastic properties of muscle and tendon. In essence, the equations describing contraction dynamics scale the isometric force predicted by the activation dynamics model. *Activation dynamics* describe the transform of the neural signal to the active state of the muscle, which corresponds to the number of actin-myosin cross-bridges. This determines the muscular tension produced without muscle shortening, i.e., the isometric force. Given the finding that the force-length dependency of contraction dynamics shifts with calcium concentration [14] which, in turn, governs activation, the computational separation of the activation and contraction dynamics may be challenged. For example, Rockenfeller and Günther recently proposed a model where muscle activation depends on the volumetric density of calcium binding sites, which in turn is a function of muscle fibre length [15,16].

The present study focuses on conceptual models of muscle activation dynamics for typical isometric force measurements (varying motoneuron firing frequency at constant muscle length). A simple and very widely used model was proposed by Zajac [13]. It applies a first-order low-pass filter to transform a rectified electromyogram (EMG) signal into a force output. The “Zajac model” is sufficient for many studies in vertebrate muscle, where muscle activation is largely determined by the number of motoneurons recruited. Other first-order models have been applied to the smooth muscle of *Aplysia* [17,18], or to the stomatogastric system of crustaceans [19]. Variants of these first-order models have also been applied to the mesothoracic extensor tibiae muscle of the stick insect *Carausius morosus* [2].

**Fig 1:**
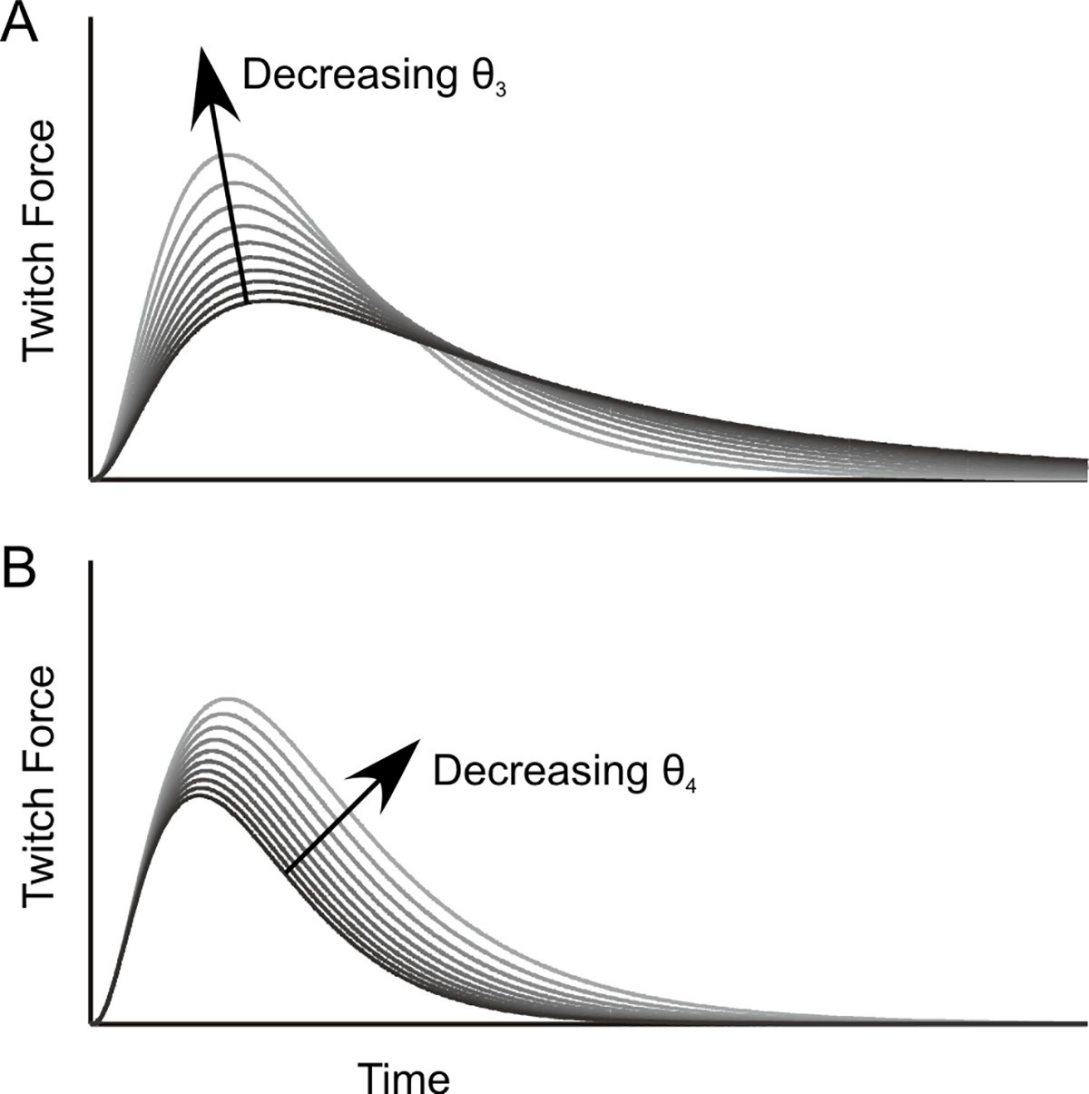
Twitch shape parameters of the Hatze-Zakotnik model. Black to grey curves show changes in single-twitch shape with variation of parameters θ_3_ (**A**) and θ_4_ (**B**) in Eq 2. Parameter θ_3_ modulates the twitch peak force and decay rate at a constant area under the twitch, whereas θ_4_ jointly modulates peak force and single-twitch duration. Zakotnik’s extension to Hatze’s original model includes a spike-frequency-dependent modulation of parameter θ_4_ (see Eq 5).

In contrast to these first-order models, models that use higher-order differential equations can create a delayed response to a pulsed input such as an action potential [20,21]. The “Hatze model” uses a pair of non-linear second-order differential equations, based on theoretical assumptions regarding the transformation of the neural signal to force [20]. Zakotnik [1] introduced a variant of this model for locust extensor tibiae muscle that includes a frequency-dependent modulation of twitch shape (Fig 1). More recently, Wilson and co-workers applied a set of different models to experimental data from the same insect muscle, finding that a linear third-order model captured well the activation dynamics following slow motoneuron stimulation [3]. Subsequently, they found that a non-linear fourth-order model was still better-suited to modelling the responses to different—slow and fast—motoneuron types [4].

Three of the five models compared in the present study, including our own former work [1] and that of Wilson [3,4], have focussed on the extensor tibiae muscle of the locust hind leg, so we have used exemplar data from that muscle for this comparative analysis. The physiology and the underlying neuronal control of this muscle have been well studied [22–25]. Time constants for force production, and descriptions of history-dependent effects such as potentiation are available [26]. The muscle consists of slow, intermediate and fast fibre types and is innervated by two excitatory motoneurons, one inhibitory motoneuron, and a neuromodulatory dorsal unpaired median (DUM) neuron [26–28]. The fast extensor tibiae motoneuron (FETi) usually fires 12 to 17 spikes at up to 70 s^-1^ as part of a stereotyped motor sequence during jumping and kicking [29,30], but is generally not active in walking [31]. It fires up to 9 spikes during each cycle of a scratching movement [32]. In contrast, the slow extensor tibiae motoneuron (SETi) is active during both fast (jumping and kicking) and slower behaviours: for example only 2 to 3 SETi spikes per step allow the muscle to produce forces sufficient for propulsion [31]. During rhythmical scratching, SETi fires bursts of up to 30 spikes at a median instantaneous rate of 96 sp s^-1^, with transient peak frequencies up to approx. 400 s^-1^ [33]. The locust metathoracic extensor tibiae muscle is thus used in a range of natural behaviours, including walking, scratching, kicking and jumping. While the hind leg is adapted for jumping, this adaptation has more to do with muscle volume, biomechanical specialisation of the joint and motor patterns than with muscle fibre physiology. Its innervation by fast, slow and common inhibitory motor neurons is the same as that of the stick insect mesothoracic extensor tibiae muscle modelled by Blümel et al. [2].

The activation dynamics model of a muscle becomes critical when the time course of force generation is not a simple function of average motoneuron spike rate. This is the case not only for the hind leg extensor muscle of locusts: but also for insect muscles in general, which are typically innervated by only a small number of motoneurons, and where single muscle potentials can generate considerable force. We present a detailed comparative evaluation of three linear and two non-linear activation dynamics models that is important for the use of insect model systems in neuroscience. We explain why non-linear models are clearly superior whenever low and intermediate spike rates up to 30 Hz are of interest; and we demonstrate how suitable the two available non-linear models are in accounting for the properties of different motoneuron and muscle types. Moreover, we present an open-source muscle activation dynamics toolbox for research and education.

## Results

Modelling realistic time courses of muscle contraction in insects requires appropriate consideration of the characteristic properties of insect muscle, including the importance of single twitches, the long-lasting twitch force, and non-linear force potentiation. As these properties may differ considerably among particular muscle activation dynamics models, we present a comparative evaluation of five models that have been proposed in the literature. We do so in four steps. First, we focus on the time course of the single twitch because it can be considered the elementary muscle contraction event. Second, we relate the properties of the single twitch to those of isometric muscle contractions in response to arbitrary motoneuron spike rates. Third, we compare and evaluate the properties of five published models. To illustrate the differences of the models in exactly the way that they were proposed originally, we initially adhere to the published parameter sets. Fourth, we evaluate the properties of the two most accurate models after optimising their parameters to one exemplar experimental data set.

### Single twitch

**Fig 2:**
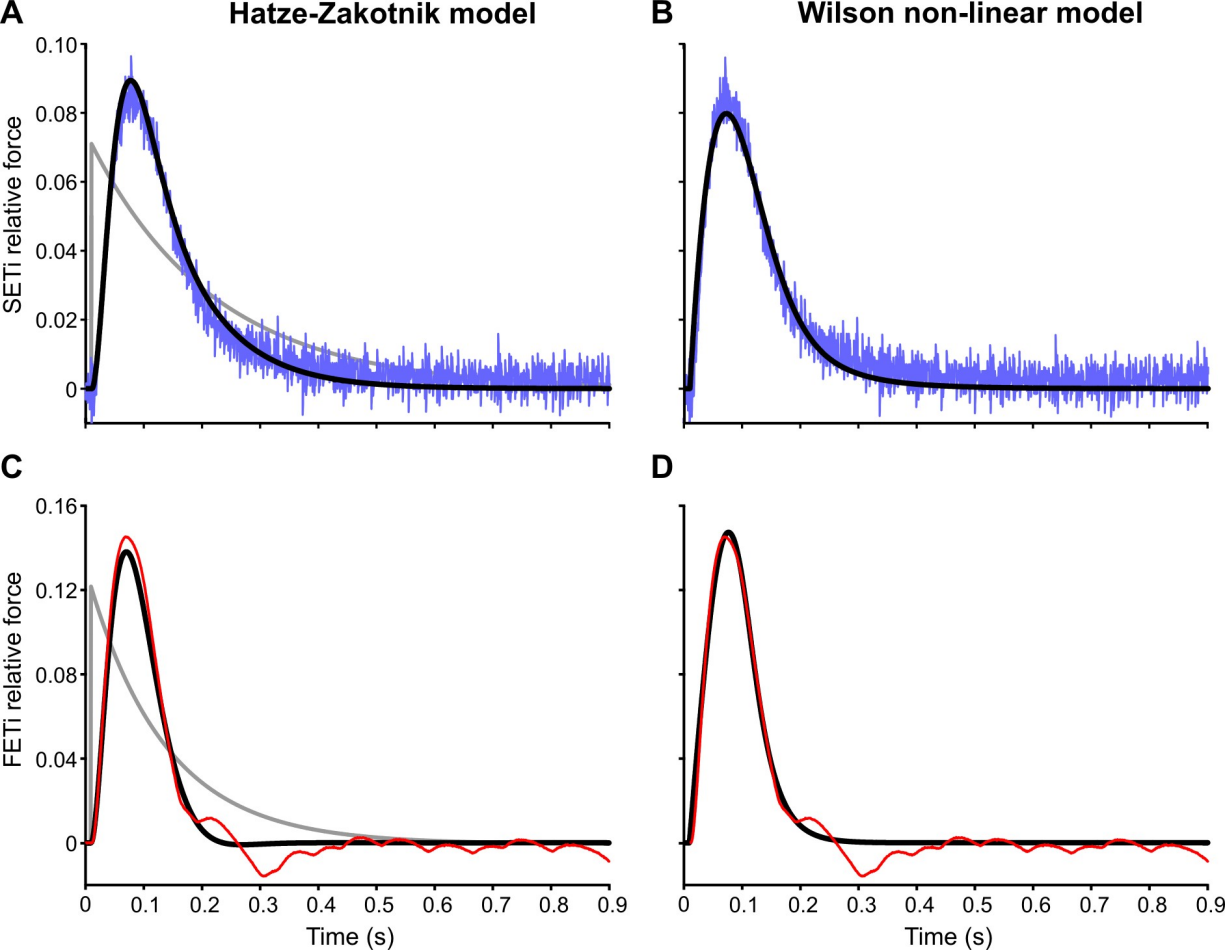
Modelling a single twitch. Top row shows an isometric twitch response to a single SETi spike (blue) and corresponding model simulation results for that single twitch (solid black) using the Hatze-Zakotnik model (**A**) and non-linear Wilson model (**B**). Force is normalised to the maximum SETi-induced force of the extensor tibiae muscle of this particular animal. The spike onset is at time t = 0. The grey lines in A and C show the force responses predicted by Zajac’s first-order model, where the rise time is much shorter than in a real twitch. **C**, **D** show an isometric twitch response to a single FETi spike (red) and corresponding simulation results (black) for the same two models. Force is normalised to the maximum FETi-induced force of the extensor tibiae muscle of this particular animal. Note that for this figure, model parameters were optimised to fit the single twitch response only. In the Hatze-Zakotnik model, there were four parameters only (parameters K1 and K2 were kept constant). In the Wilson non-linear model, there were six.

Single twitch contractions of insect muscle are generally fairly slow: for both slow and fast motoneurons, a single action potential may lead to a change in isometric muscle force for 200 ms or longer (Fig 2). In response to an SETi action potential (Fig 2A and 2B), the time course of force development in the extensor tibiae muscle was of sigmoid shape with maximum rising velocity at approximately 25 ms. The time to peak force was on average 67 ms (SD: 9 ms) so force development was always faster than force decay, which could take up to 1 s. In response to an FETi action potential (Fig 2C and 2D) the overall shape of the isometric force time course was similar to that following an SETi action potential, but peak force was considerably higher. The time to peak force was on average 61 ms (SD: 6 ms). In some experiments on FETi stimulation, a single stimulus caused additional weak contractions of unknown origin that followed the initial strong twitch (Fig 2C and 2D).

In Fig 2, both the Hatze-Zakotnik and the non-linear Wilson models were fitted to experimental recordings of single twitches. In the case of the Hatze-Zakotnik model, this involved only four parameters (θ_1_-θ_4_) because no parameters were needed to model force potentiation (see Eqs 1, 4 and 6: c(f) = 1 for f = 1, irrespective of K_1_ and K_2_). In contrast, Wilson's model requires optimisation of six parameters. Both models achieved very good fits. For example, the Hatze-Zakotnik model accurately captured single twitch force time courses with an average root mean square error (RMSE) of 1.1% of peak twitch force for SETi stimulation, and 6.6% for FETi stimulation (solid black lines in Fig 2). For comparison, modelling the twitch response with a first-order model (e.g., [13]) leads to an exceedingly short rise time and lacks the rounded peak observed in experiments (dashed lines in Fig 2). The third-order, linear Wilson model can reproduce the delayed, sigmoid increase and rounded peak [3].

Modelling force potentiation (i.e., the supra-linear increase of peak force with increasing stimulation frequency) requires a non-linearity. One way to incorporate such a non-linearity is to alter the shape of a single twitch in a frequency-dependent manner. In the case of Hatze's model (Eqs 1 and 2), this can be achieved by varying parameters that affect not only the peak twitch force, but also the rates of increase and decay (θ_3_) or the duration of the twitch (θ_4,_ see Fig 1). In the Hatze-Zakotnik model, θ_4_ is modified by the frequency-dependent factor c(f)). As a result, summation of twitches is adjusted so as to capture frequency-dependent force potentiation.

### Hatze’s model and its variants

**Fig 3:**
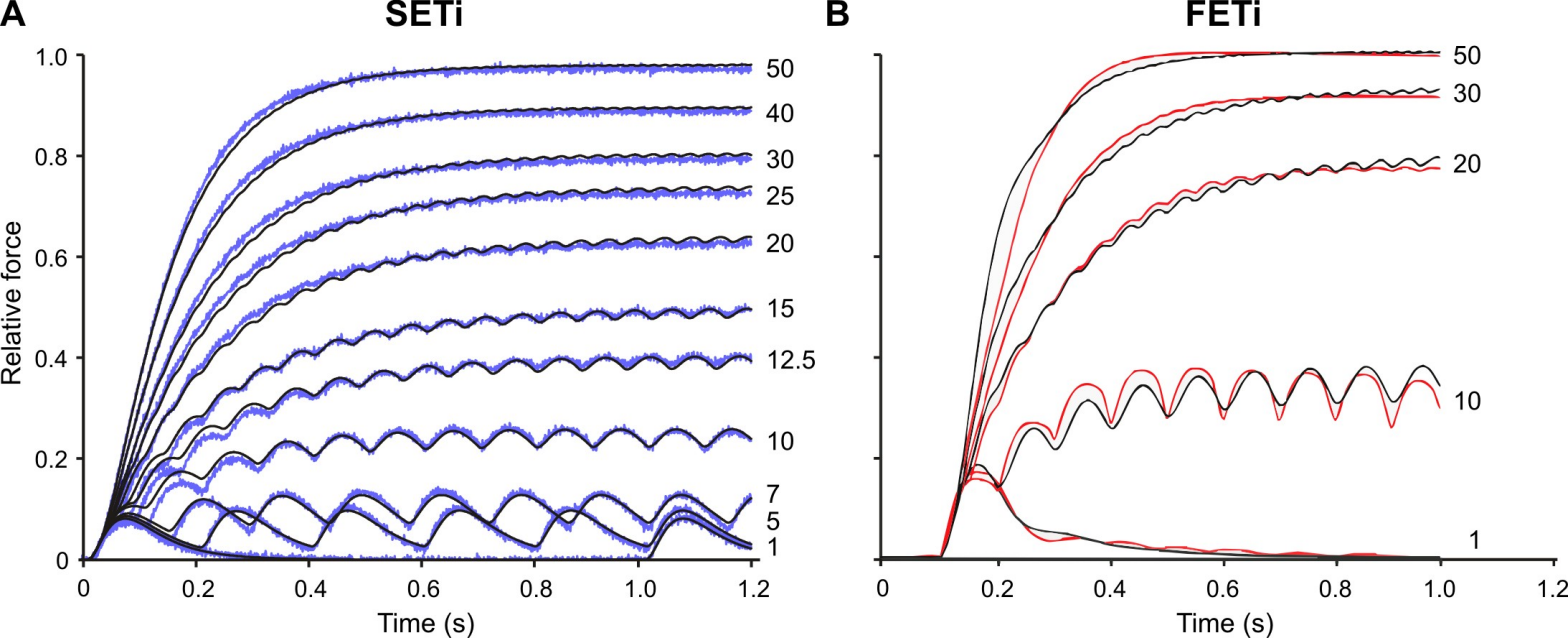
Frequency-dependent modulation of single twitch shape can explain non-linear force potentiation. Measured isometric force traces in response to SETi (blue, **A**) and FETi stimulation (red, **B**) and corresponding simulation using Hatze-Zakotnik model (black). Stimulation frequencies (Hz) are indicated to the right. Parameters θ_1_ - θ_4_ were optimised for the single twitch (as in Fig 2A and 2C) and then θ_4_ was optimized separately for each spike frequency. Each SETi time course could be fitted extremely well except for the first three twitches at 10 and 12.5 Hz. For FETi, the model slightly overestimates the rise time at high frequencies and underestimates the rise time at low frequencies.

The effect of non-linear force potentiation is best observed in the Hatze-Zakotnik model when increasing the motoneuron spike rate from 10 Hz to 50 Hz, for both slow (SETi) and fast (FETi) motoneuron stimulation (Fig 3). The increase in peak force was much larger when the stimulus frequency was changed from 10 Hz to 20 Hz than when it was changed from 20 Hz to 50 Hz. At stimulus frequencies above 20 Hz, single twitches fused into a smooth tetanic contraction for both SETi (Fig 3A) and FETi (Fig 3B).

Two extensions of Hatze’s model have been proposed to account for non-linear force potentiation. For vertebrate muscle, van Zandwijk and colleagues [34] suggested scaling the peak twitch force by a sigmoid function. However, application of this Hatze-van-Zandwijk model to the insect data shown in Fig 3 proves insufficient. S2 Fig shows the result after optimising the sigmoid function parameters A and γ_0_ of Eq 3 by fitting force traces of all stimulation frequencies with constant coefficients θ_1_ to θ_4_. While the Hatze-van-Zandwijk model performs reasonably well for low stimulation frequencies (S2 Fig), it fails to replicate the experimental data for higher stimulation frequencies. The time taken to reach maximum tetanic force is shorter in the model than in experiments. For example, a full tetanus for SETi stimulation at 50 Hz is generated after approximately 300 ms in the model, but only after 600 ms in the experiment. In addition, force values in the model are too low at SETi frequencies above 10 Hz. Furthermore, the tetanus fuses only at frequencies above 25 Hz.

**Fig 4:**
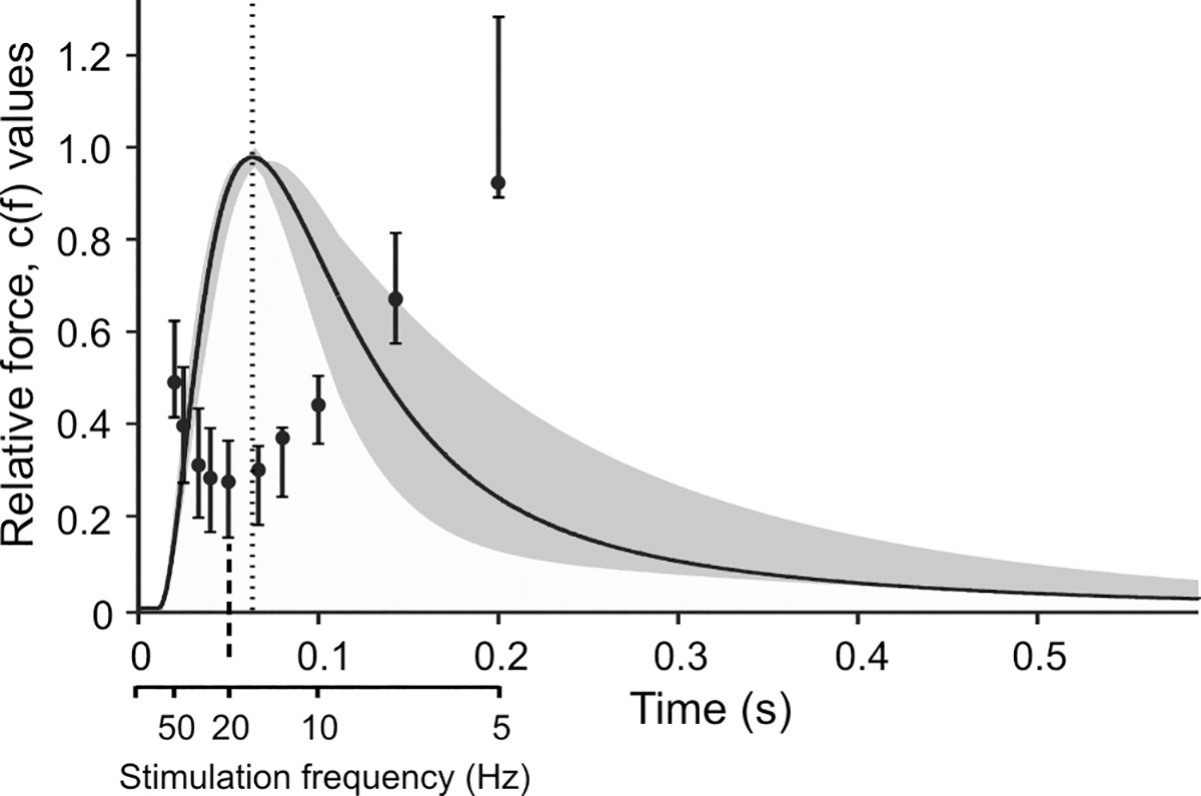
Frequency-dependent scaling of single twitch force. Values of function c(t) of Eq 6 for different SETi stimulation frequencies. Black circles and solid vertical lines indicate medians and inter-quartile ranges. Smaller values indicate a stronger potentiation of twitches, with a minimum at 20 Hz (dashed line). The smaller this value, the stronger is force potentiation (see Fig 1). As a reference, the values are superimposed on a single twitch (black curve and shaded area indicate mean and inter-quartile range of experimental data, n = 5). Maximum potentiation was achieved when a spike occurred approximately 15 ms before peak twitch force generated by the preceding spike (displacement of dashed and dotted lines). The lower scale relates frequency to inter-spike interval because in Eq 5 factor c is a function of frequency, whereas in Eq 6 it is a function of time.

A much better fit to experimental data was obtained when the sigmoid relationship was replaced with a non-linear, frequency-dependent force potentiation. Fig 3 shows a direct comparison of measured and simulated force profiles for a representative SETi stimulation experiment, with a constant set of parameters θ_1_ to θ_3_ (optimised to single twitch) and frequency-specific optimisation of parameter θ_4_ (see Eq 4). The resulting model predictions match the experimental data extremely well across all stimulation frequencies. Fig 3 thus demonstrates the concept of the Hatze-Zakotnik model that scales parameter θ_4_ by the frequency-dependent factor c(f) (Eq 5). Table 1 lists the improvement achieved by optimisation of θ_4_. The fit quality improved in all cases tested. Although additional optimisation of θ_3_ improved fit quality substantially in one test data set, it led to much less improvement or even lead to worse results in other cases. To show how factor θ_4_ needs to be modulated with spike frequency, Fig 4 plots the optimal values of c(f) relative to the inter-spike interval 1/f, superimposed on the time course of a single twitch. Potentiation factor c(f) is 1 for a single twitch (i.e., no potentiation) and less than 1 for higher stimulus frequencies. For both SETi and FETi stimulation, c(f) was lowest at an inter-spike interval of 0.05 s, equivalent to f = 20 Hz (dotted line in Fig 4). The overall shape of the function graph c(1/f) resembles the shape of an inverted twitch, with its minimum approximately 15 ms before peak twitch force. During a spike train, force potentiation is thus largest if a spike occurs just prior to the maximum twitch force caused by the preceding spike. In the experimental data, this effect was consistent across all preparations.

**Table 1:** RMS error values for three kinds of optimization runs for the Hatze-Zakotnik model prediction for SETi data (11 frequencies of 1 s duration from each animal). Optimising θ_4_ (middle column) always improves fit quality compared to the reference parameters of Zakotnik (2006). Additional optimization of θ_3_ (right column) may or may not further improve fit quality.

Animal	Fixed—θ_1_, θ_2_, θ_3_, θ_4_, K_1_ and K_2_	Fixed—θ_1_, θ_2_, θ_3_ Varying—θ_4_	Fixed—θ_1_, θ_2_ Varying—θ_3_, θ_4_
A	0.0925	0.0643	0.0345
B	0.2327	0.2188	0.2225
C	0.5224	0.5198	0.0719
D	0.0605	0.0256	0.0224

**Fig 5:**
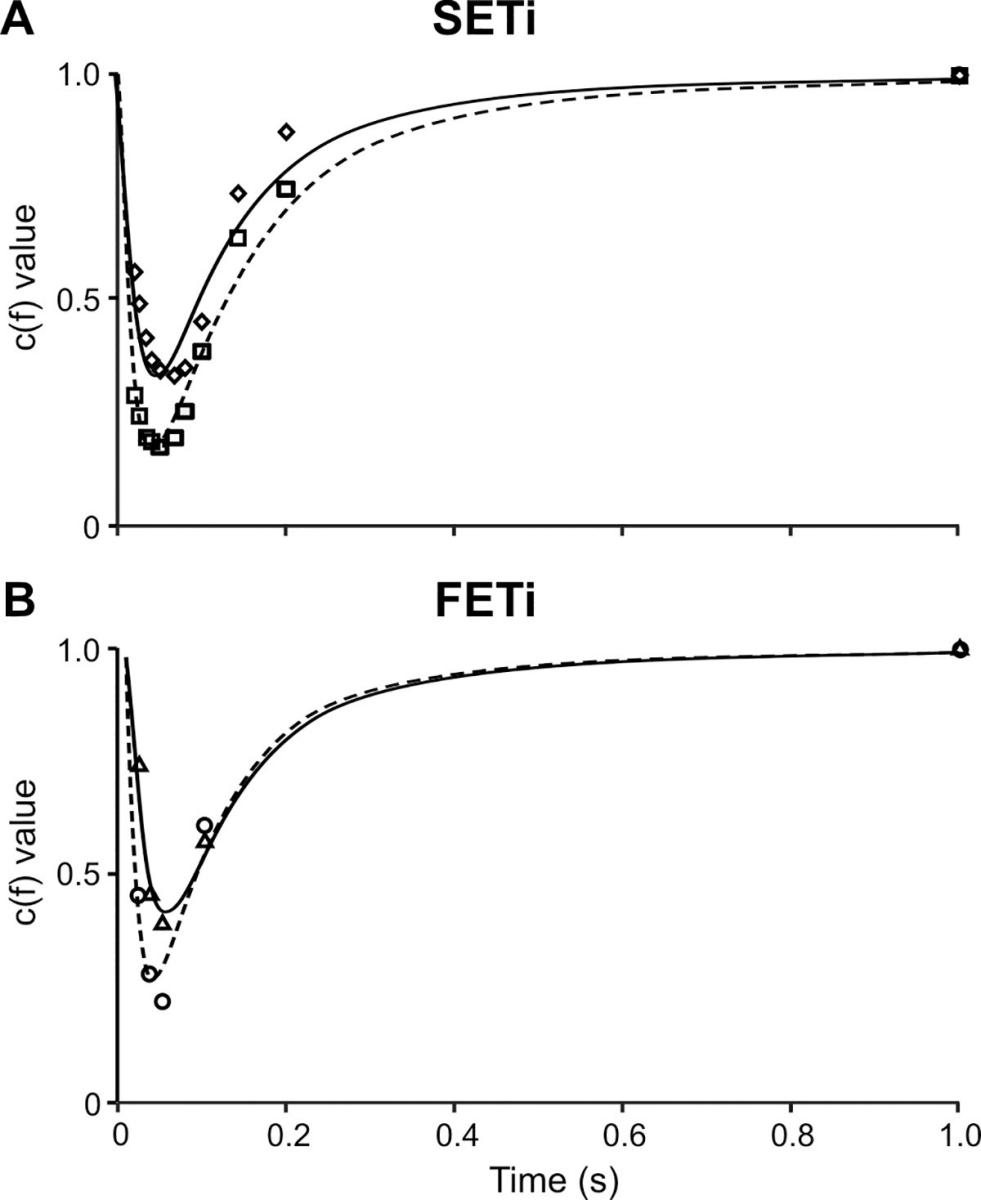
Values of force potentiation factor c as a function of inter-spike interval. Both SETi (**A**) and FETi (**B**) panels show data from two animals (different symbols for different animals). For each symbol, factor c was calculated after frequency-specific optimisation of θ_4_ to measured force traces, as shown in Fig 3. Fits are Michaelis-Menten-type functions according to Eq 6 (solid and dashed lines). Each function fit was weighted by the stimulus frequency, improving fit quality at small inter-spike intervals.

The frequency-dependent potentiation factor c(f) in the Hatze-Zakotnik model is implemented as a Michaelis-Menten function (Eq 6). The two parameters of this function were obtained by least-square fits to the experimental data from four preparations (two SETi, and two FETi stimulation experiments). As shown in Fig 5, Eq 6 approximated well the determined values for c: it was 1 for a single twitch (equivalent to t = 1 s) and reached a minimum at about t = 0.04s (24.9 Hz). Mean parameter values for SETi were K_1_ = 1.46·10^−2^ (SD: 2.3·10^−3^) and K_2_ = 3.9·10^−4^ (SD: 2.6·10^−4^). For FETi, mean parameter values were K_1_ = 7.8·10^−2^ (SD: 2.6·10^−2^) and K_2_ = 8.0·10^−4^ (SD: 1.8·10^−4^). For an overview of all model parameters see Table 2.

**Table 2:** Estimated model parameters for six locusts when fitting to SETi data (animals A, B, and D) or FETi data (animals 2, 3, and 4), using both the Hatze-Zakotnik and Wilson non-linear models. Published model parameters are shown for comparison.

	Model	Hatze-Zakotnik Model							Wilson non-linear Model						
Motoneuron	Locust	θ_1_	θ_2_	θ_3_	θ_4_	K_1_	K_2_	Error	τ_c_	τ_1_	τ_2_	k	A	m	Error
**SETi**	A	101	2.956	1.858	34.666	1.77e-2	8.37e-4	0.0877	0.088	0.095	-0.027	1.45	47.65	2.34	0.0491
	B	105	4.970	3.697	61.239	1.05e-2	2.03e-4	0.1693	0.059	0.186	-0.210	2.33	48.42	2.59	0.1119
	D	72	2.019	6.770	70.464	1.30e-2	7.04e-4	0.0498	0.152	0.005	0.103	1.57	25.59	1.59	0.0400
	Zakotnik *et al*. 2006, mean	79	2783	4919	78.582	1.46e-2	3.90e-4	-	-	-	-	-	-	-	-
	Wilson *et al*. 2013, mean	-	-	-	-	-	-	-	0.11	0.05	0.00	6.55	24.39	1.91	-
**FETi**	2	28	1696	9.303	71978	4.70e-2	2.1e-3	0.2706	0.072	0.013	0.080	0.77	19.31	3.05	0.0560
	3	34	1687	5.385	37357	0.76e-2	2.6e-3	0.4788	0.093	0.067	0.040	1.05	25.74	2.16	0.0509
	4	51	1010	3.577	120970	1.06e-2	7.0e-4	0.1582	0.083	0.069	0.019	1.08	30.12	3.37	0.0525
	Wilson *et al*. 2013, mean	-	-	-	-	-	-	-	0.070	0.083	0.10	0.57	5.8	1.8	-

### Comparison of published models

**Fig 6:**
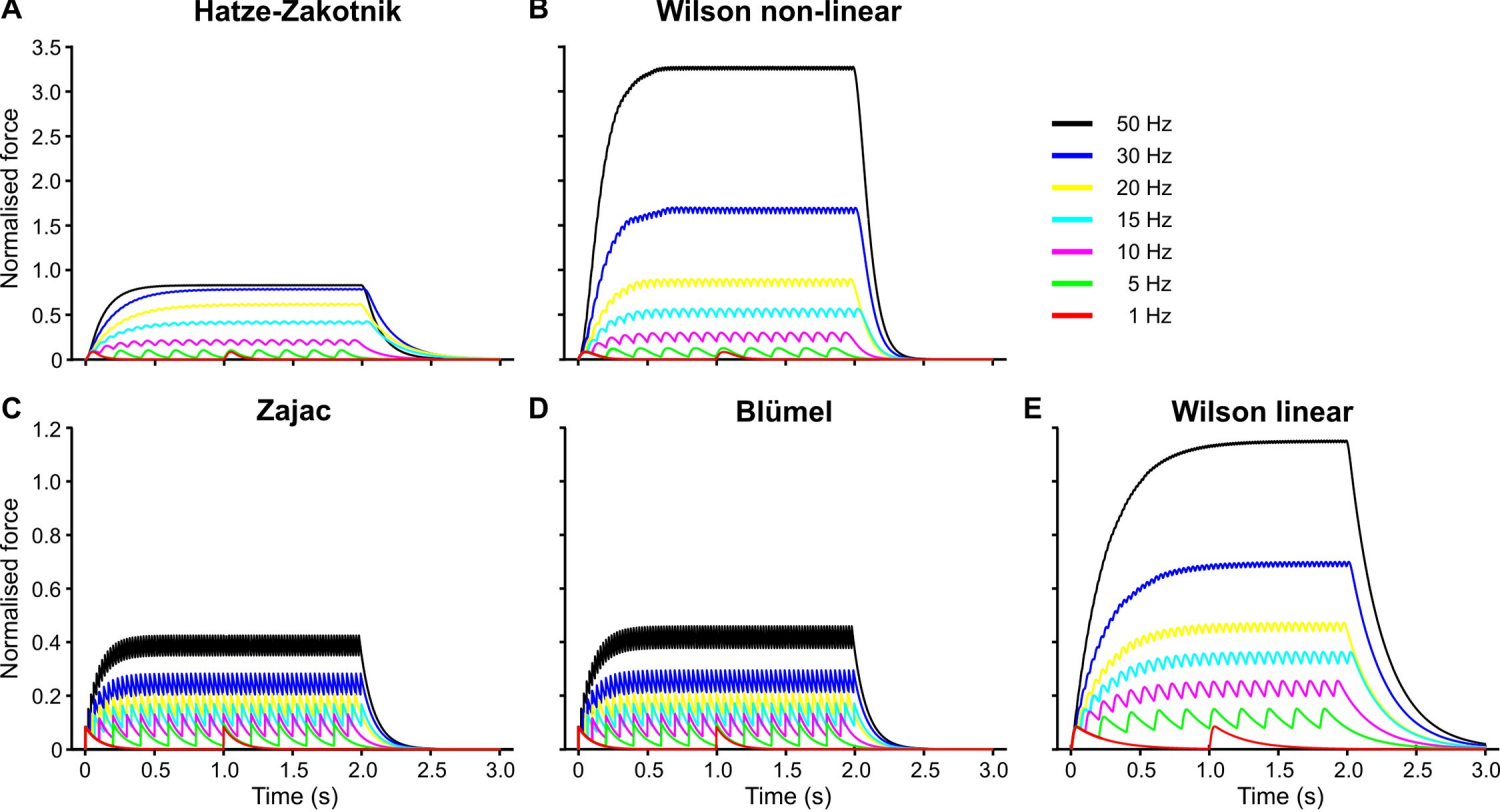
Constant frequency responses of five muscle activation models “as published”. **A**, **B** show the time course of isometric SETi contractions at different stimulation frequencies (1–50 Hz) for two kinds of second-order, non-linear models: the Hatze-Zakotnik model [65] and the non-linear Wilson model ([4]. Note that, for immediate comparison, model output was normalised to maximum force of the single-twitch. This was set to 0.1. **C-E** show corresponding time courses of three published linear models: (**C**) [13], (**D**) [2] (both first order), and (**E**) [3] (third order). Constant frequency stimulation started at t = 0 s and persisted for 2 s. For comparison with FETi contractions see S4 Fig.

**Fig 7:**
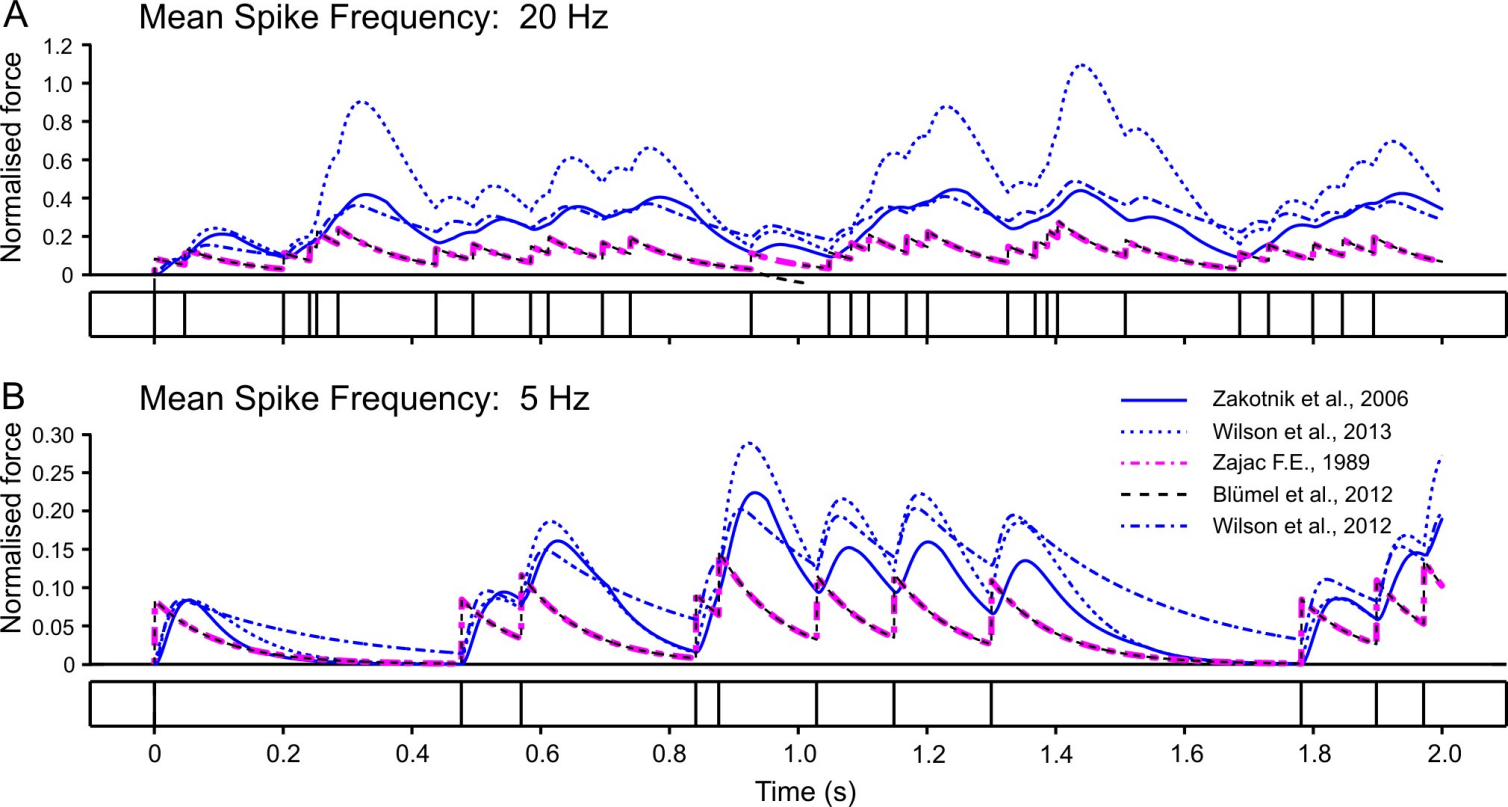
Response to random activation. Comparison of simulated isometric SETi contraction forces in response to two Poisson spike trains with mean frequencies of 20 Hz (**A**) and 5 Hz (**B**). The same models and model parameters are used as in Fig 6. Differences between models are most prominent where force potentiation is strongest, i.e., when inter-spike intervals are approximately 50 ms. The time courses predicted by the Hatze-Zakotnik and linear Wilson models are relatively similar, as are those of the two first-order models. The non-linear Wilson model deviates most strongly from the others. For comparison with FETi contractions see S5 Fig.

**Fig 8:**
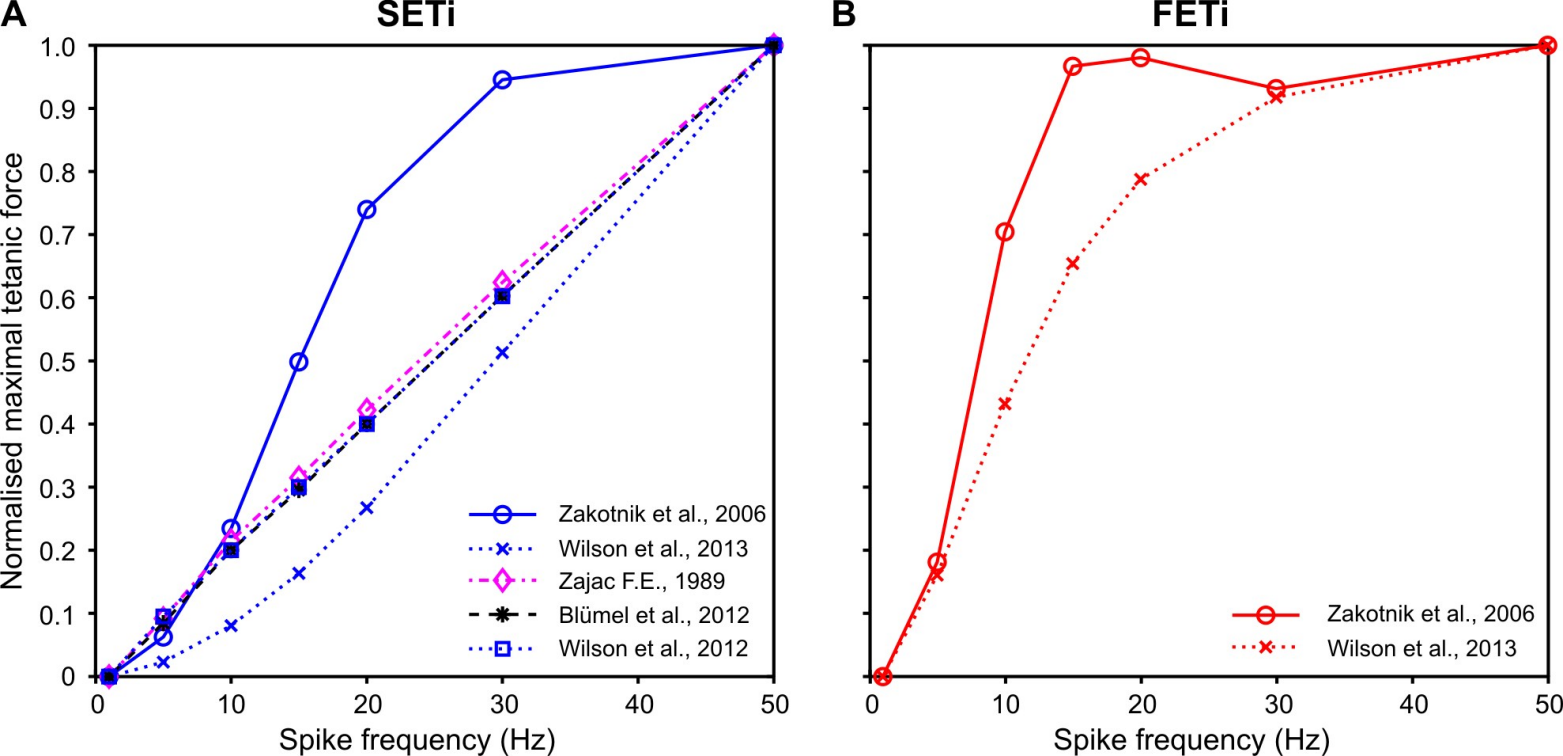
Frequency dependence of peak isometric force. As a summary of Fig 6 (**A:** SETi) and S4 Fig (**B:** FETi), normalised peak isometric force was plotted as a function of spike frequency for constant stimulation. Data were normalised to the peak force for a stimulation frequency of 50 Hz. For linear models, peak force linearly depends on stimulation frequency. With the published parameter sets, the Hatze-Zakotnik model has a saturating, supra-linear non-linearity for SETi stimulation, whereas the non-linear Wilson model has a sub-linear non-linearity. For FETi stimulation, both models are supra-linear, with stronger saturation for the Hatze-Zakotnik model.

**Fig 9:**
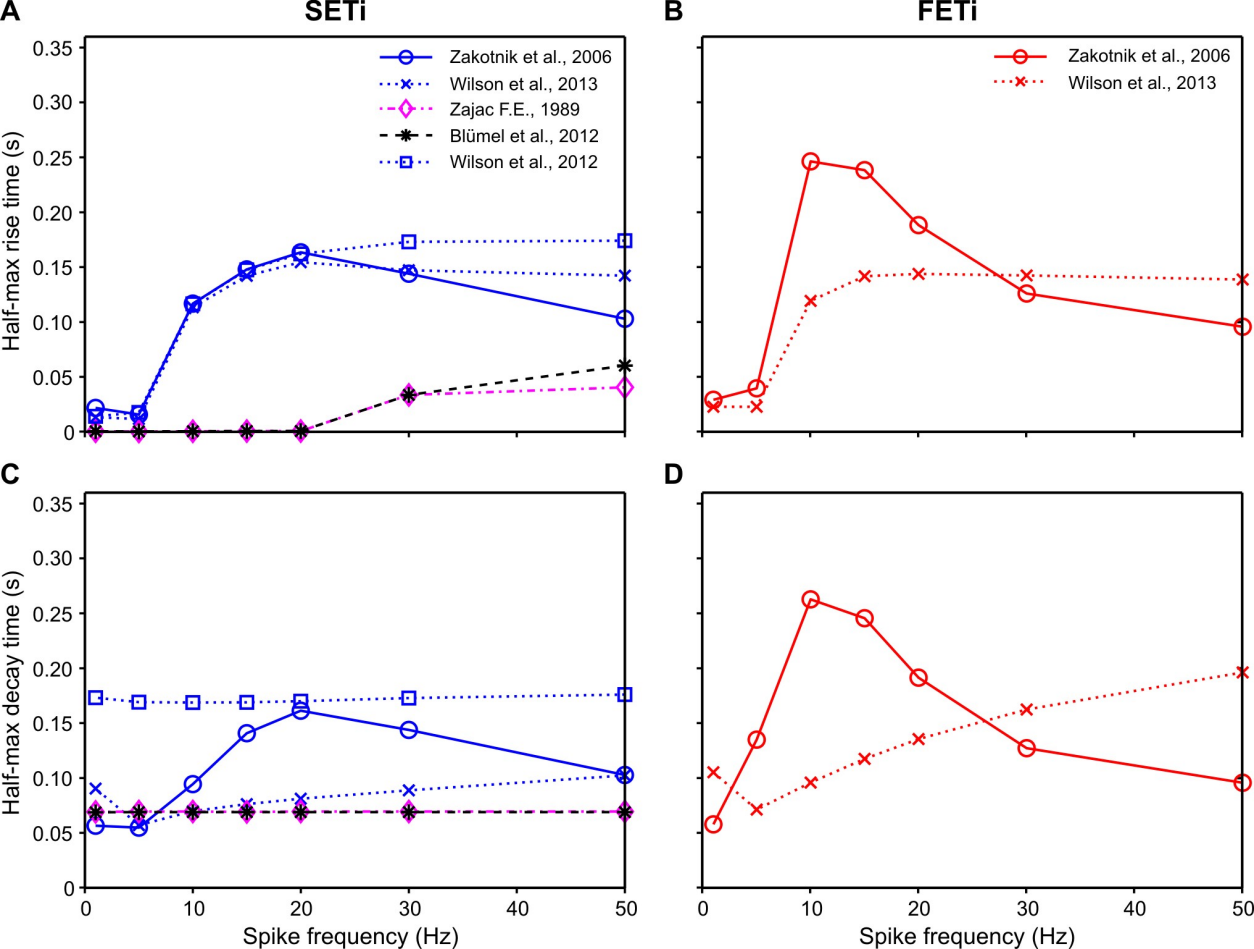
Half-maximal rise and decay times. Rise time to 50% of peak force at a given constant stimulation frequency for SETi (**A**) and FETi (**B**) is shown in the top row. **C, D:** Decay time from peak to 50% of peak force for SETi and FETi, respectively. The results were derived for the same models as used in Figs 6–8. Linear models show no frequency-dependence of decay time, and only the third-order Wilson linear model has frequency-dependent rise time. The two non-linear models differ most strongly with regard to their decay time, particularly for FETi stimulation.

To quantitatively compare the properties of available muscle activation models “as published”, we examined their responses to constant-frequency stimulation of the slow motoneuron SETi (Fig 6, normalised to single twitch force), the corresponding responses to more natural random spike trains (Fig 7), the dependence of maximum isometric force on stimulation frequency (Fig 8A), and the rise and decay rates in response to a step onset or offset of stimulation (Fig 9A and 9C). Additionally, we compared the properties of the two non-linear models with parameter sets for a fast motoneuron (constant-frequency stimulation: S4 Fig; random stimulation: S5 Fig; peak force: Fig 8B; rise and decay rates: Fig 9B). Note that these comparisons were based on the parameter sets that were published in the original model descriptions. Accordingly, differences may, to some extent, depend on the particular properties of the experimental data sets that had been used to obtain these parameters sets in the first place.

The two first-order linear models (Zajac and Blümel) stand out in three ways: first, they cannot replicate the typical rounded peaks of muscle twitches (Fig 6C and 6D; Fig 7); second, they imply perfectly linear force potentiation with increasing spike frequency (Fig 8A); third, they show very little frequency-dependence in rise time to half-maximal force (Fig 9A)—and none at all for decay time (Fig 9C). With regard to all of these properties, they clearly differ from the properties of muscle. Examining the properties of the third-order linear Wilson model shows that some of these short-comings are a consequence of the first-order dynamics of the Blümel and Zajac models, whereas others are a consequence of linearity. For example, the linear Wilson model produces natural looking rounded twitch peaks (Fig 6E; Fig 7) and shows a strong change of rise time for stimulation frequencies between 10 and 20 Hz (Fig 9A). Both of these properties are related to the third-order dynamics of the model. In contrast, the linear Wilson model behaves the same as the other linear models in that it has perfectly linear force potentiation (Fig 8A) and lacks frequency-dependence of decay time (Fig 9C). Only the non-linear muscle activation models can capture the latter two properties of insect muscle.

With their parameter settings “as published”, the two non-linear models have considerably different properties. For example, the tetanic force for 50 Hz stimulation of a slow motoneuron reached more than thirty times the peak force of a single twitch in the non-linear Wilson model (Fig 6B), whereas it was less than ten times peak twitch force in the Hatze-Zakotnik model (Fig 6A). Differences between models are most prominent where force potentiation is strongest, i.e., when the spike frequency is around 20 Hz (Fig 7A). This is very similar for both the SETi stimulation (Fig 6) and the FETi stimulation (S4 Fig). The difference is reflected in a supra-linear (saturating) frequency dependence of maximum isometric force for the Hatze-Zakotnik model, and a sub-linear dependence for the non-linear Wilson model (Fig 8). Note that in the linear force-potentiation range (i.e., at low stimulation frequencies), the linear Wilson model behaves in a similar way to the non-linear Hatze-Zakotnik model (Fig 7A). For random FETi stimulation, the overall time courses of the two non-linear models were similar, though with considerably higher peak force for the Hatze-Zakotnik model (S5 Fig).

Although all of the higher-order models have a very similar frequency-dependence of half-maximal rise time (Fig 9A), they differ strongly in their frequency-dependence of decay time. At the end of a spike train of either SETi or FETi, measured extensor tibiae muscle force decayed in an exponential manner (S6 Fig). Similarly, all muscle activation models show an exponential decay after stimulus offset (Fig 6). The half-maximal decay time of the Hatze-Zakotnik model is strongly frequency-dependent (Fig 9C and 9D), whereas in the non-linear Wilson model it is hardly frequency-dependent in the case of SETi stimulation (Fig 9C), and shows nearly opposite behaviour to the Hatze-Zakotnik model for FETi stimulation (Fig 9D). In the latter case, decay time peaked at 10 Hz and then decreased with increasing frequency in the Hatze-Zakotnik model, whereas it reached a minimum at 5 Hz and then increased with increasing frequency in the non-linear Wilson model.

Finally, the tested muscle activation models differ strongly with regard to computational efficiency (Table 3). Whereas the two linear-first order models clearly outperform all other models, the third most efficient model is the iterative solution of the Hatze-Zakotnik model. It is only fifteen times slower than the linear first-order models, more than ten times faster than the linear Wilson model and almost 50 times faster than the non-linear Wilson model.

**Table 3:** Comparison of maximum CPU-time for simulating a 40 Hz spike train of 2 s followed by a relaxation time of 1 s (n = 10). The iterative version of the Hatze-Zakotnik model is only fifteen times slower than the linear first-order models, and nearly 50 times faster than the non-linear Wilson model.

Model	Hatze-Zakotnik model		Wilson non-linear model	Zajac linear model	Blümel linear model	Wilson linear model
	Iterative	ODE				
CPU-time (s)	0.065	1.321	3.156	0.004	0.003	0.838

### Model comparison with same experimental data

Given the considerable differences among the published models, it was important to determine the extent to which these differences are due to the computational properties of the models, or rather due to differences in the experimental data for which the published parameter sets had been optimised. To resolve this issue, we fitted the two non-linear models to the same experimental data set, obtaining three particular solutions per model for both SETi and FETi stimulation (see Table 2 for model parameters and performance measures). Owing to the limitations of the linear models, as mentioned in the previous section, we did not consider them further.

**Fig 10:**
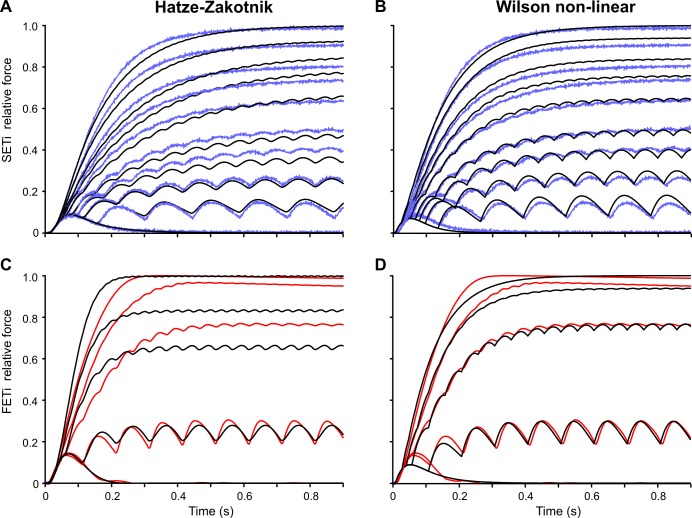
Comparison of non-linear models optimised to the same experimental data. Model fits (black) to experimental data sets for SETi (**A, B**, blue) and FETi (**C, D**, red) stimulation. Both models have six free parameters. Since the non-linear Wilson model optimises the complete parameter set for all force traces simultaneously, its single-twitch fit is worse than that of the Hatze-Zakotnik model. In the latter, four parameters are optimised for the single twitch, and the remaining two describe the frequency-dependent modulation of the single-twitch time course. Constant stimulation frequencies used were: 1, 7, 10, 12.5, 15, 20, 25, 30, 40 and 50 Hz for SETi, and 1, 10, 20, 30 and 50 Hz for FETi.

**Fig 11:**
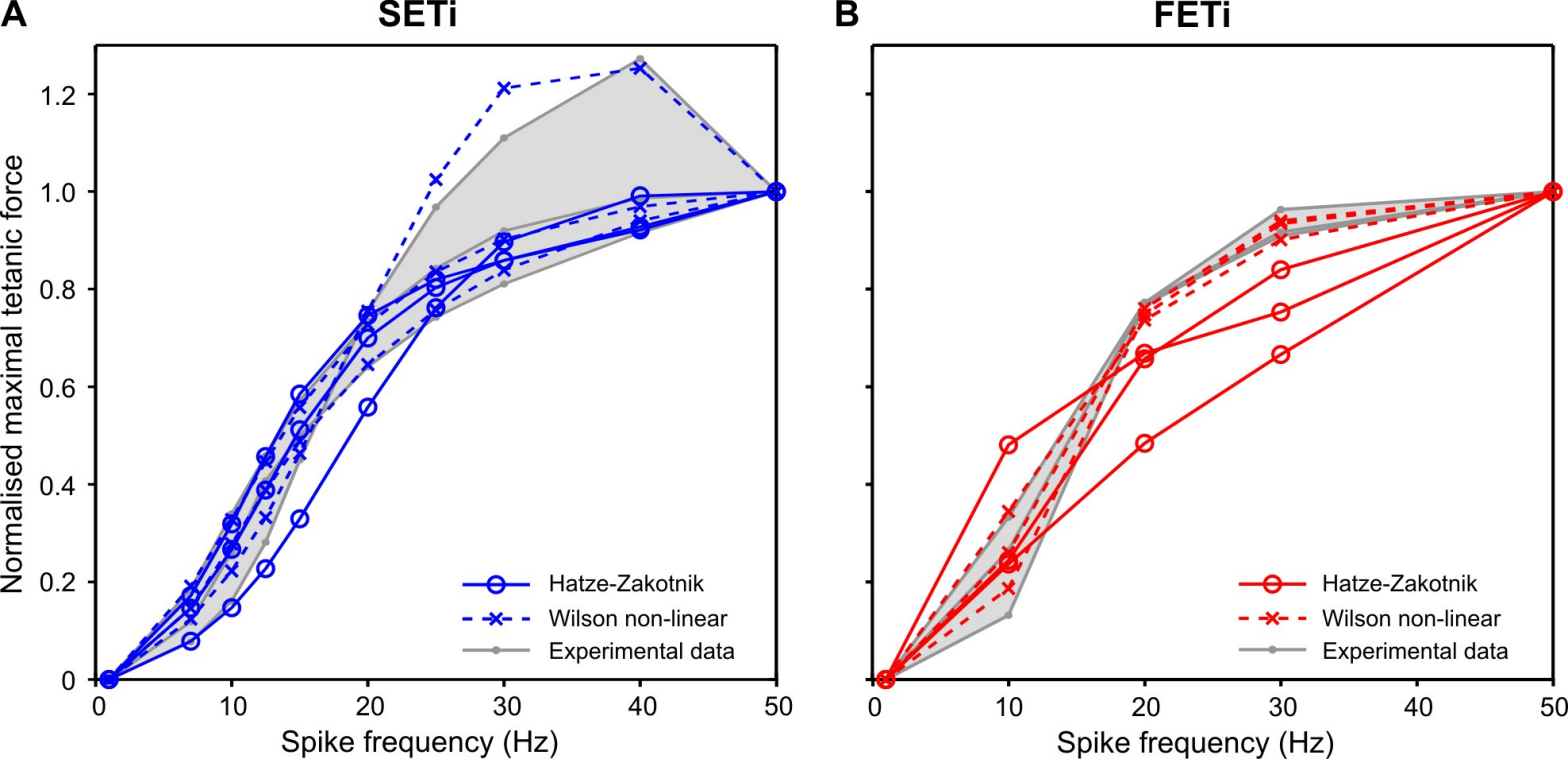
Frequency-dependent force potentiation. Both non-linear models were fit to data from six preparations, three for SETi (**A**) and three for FETi (**B**). The experimental data range (N = 3) is shown in grey. Values were normalised to peak force obtained for stimulation at 50 Hz.

The overall fit quality of the two models is shown in Fig 10. Both models can simulate isometric force time courses similarly well for SETi stimulation (Fig 10A and 10B). The core of the Hatze-Zakotnik model is an accurate model of a single twitch response that is then modulated in a frequency-dependent manner, so the model’s fit quality is best for low stimulation frequencies. With increasing frequency it tends to overestimate the half-maximal rise time, such that force build-up is slightly slower than in the experimental data (Fig 10A). In contrast, parameters of the non-linear Wilson model are optimised across all spike frequencies simultaneously, such that fit quality is similar for all stimulation frequencies. As a consequence, the fits to the single twitch and to responses to low-frequency stimulation are less good than those of the Hatze-Zakotnik model. For intermediate SETi stimulation frequencies, the non-linear Wilson model tends to underestimate rise time (Fig 10B). With regard to FETi stimulation, the Hatze-Zakotnik model performs less well than for SETi stimulation. It underestimates both the rise time and the maximum force for intermediate stimulation frequencies (Fig 10C). In comparison, the non-linear Wilson model achieves a better fit of the maximum force, while slightly overestimating rise time (Fig 10D). When comparing fit quality across experimental data sets, both non-linear models prove to capture the frequency-dependent force potentiation equally well for SETi stimulation (Fig 11A). Regarding FETi stimulation, the non-linear Wilson model achieves better fits (Fig 11B, compare model results with shaded area of experimental data). Despite the fact that the Hatze-Zakotnik model could replicate the saturating force potentiation curves well for SETi, optimisation did not lead to a similarly good match for FETi stimulation.

**Fig 12:**
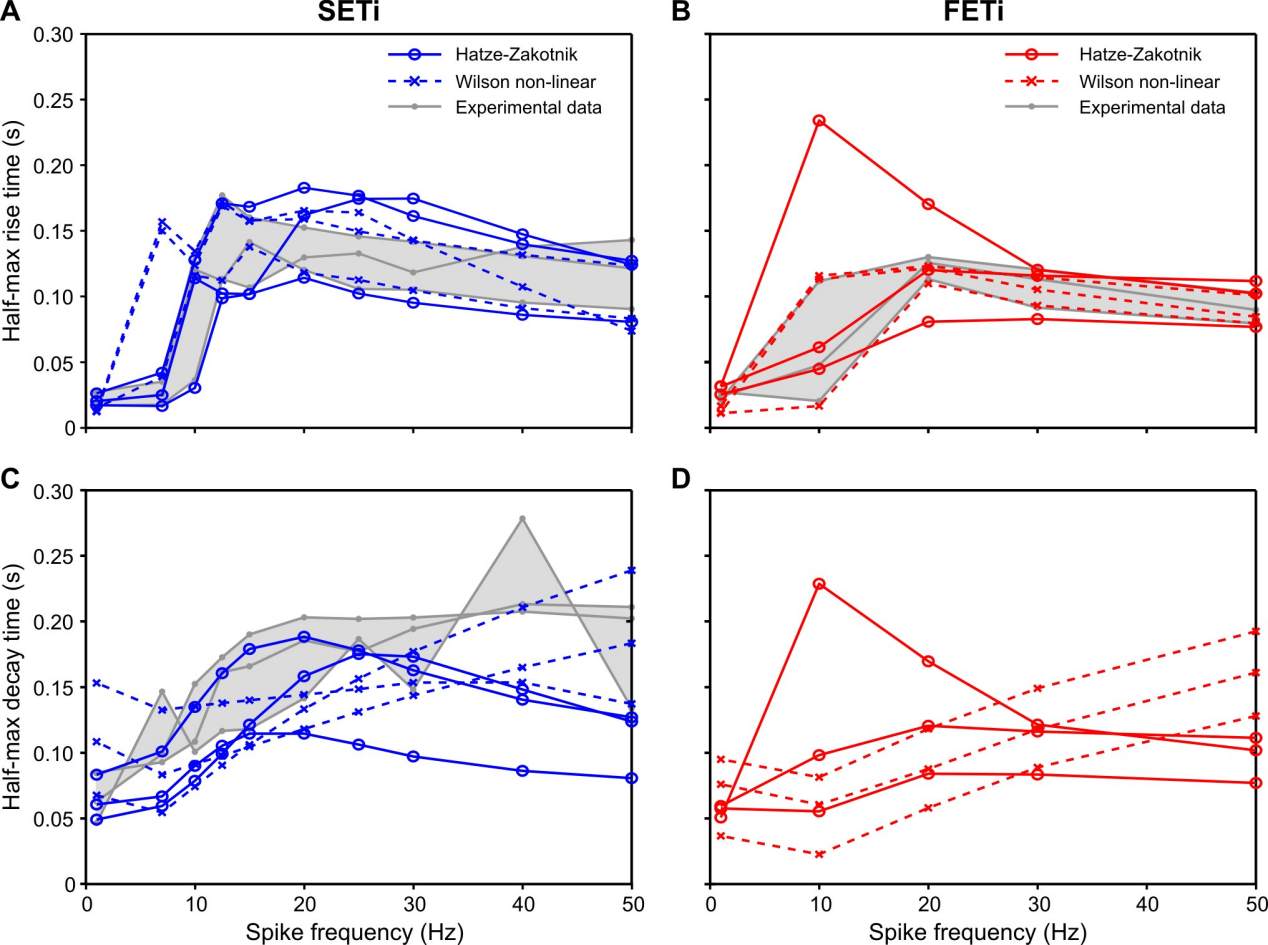
Half-maximal rise and decay times. **A, B**: Rise time to 50% peak force at a given constant stimulation frequency for SETi (**A**) and FETi (**B**). **C, D**: Decay time from peak to 50% peak force for SETi (**C**) and FETi (**D**). Both models were fit to the same experimental data sets as used for Fig 11. The experimental data range is shown in grey (N = 3). No experimental data are available for frequency dependence of FETi decay. Continuous and dashed colour lines depict best-fit results for the Hatze-Zakotnik and non-linear Wilson models, respectively.

As described above for the models ‘as published’ (Fig 9), frequency-dependent change of half-maximal rise time is similar in both optimised models (Fig 12A: SETi, 12B: FETi). For low frequency stimulation of two SETi data sets, the non-linear Wilson model overestimated rise time by more than 100 ms (Fig 12A). The Hatze-Zakotnik model overestimated rise time to a similar degree for one FETi data set (Fig 12B). Concerning the half-maximal decay time, the two models differ in much the same way as described for Fig 9. Whereas the Hatze-Zakotnik model shows the longest decay times for low to medium stimulation frequencies, with faster decay after high-frequency stimulation (Fig 12C and 12D), five parameter sets of the non-linear Wilson model lead to increasingly slower decay with increasing stimulation frequency. When comparing the models with experimental data, the Hatze-Zakotnik model underestimated decay time at high frequencies, whereas the non-linear Wilson model underestimated decay at intermediate frequencies of SETi stimulation (Fig 12C). S6 Fig compares the decay time courses of the Hatze-Zakotnik model with experimental data. For SETi stimulation, the model fit is very good for low frequencies (5 and 10 Hz). At higher stimulation frequencies (20 and 40 Hz) the main difference between model and experimental data was the delayed onset of decay in the experiment. As a consequence, the decay time course of the model looks very similar to the experimental time course, but the former leads the latter by approximately 70 ms. For FETi stimulation, the model differed from the experimental data in two ways: for stimulation at 50 Hz, the experimental decay again lagged the model decay; the decay of the experimental data was also considerably slower than in the model for stimulation at 10 and 20 Hz (S6 Fig).

In summary, the non-linear models are similarly capable of replicating the properties of slow motoneuron (SETi) induced isometric contraction, with the Hatze-Zakotnik model being slightly superior with regard to the half-maximal decay time. For stimulation of the fast motoneuron (FETi), the non-linear Wilson model achieves better fits, particularly with regard to frequency-dependent force potentiation.

## Discussion

The choice of an appropriate muscle activation model is important whenever dynamic properties of movement sequences are of interest. In the simplest case, muscle activation is a function of the number of motoneurons recruited and can be related to the overall instantaneous firing rate. This is the rule in vertebrates, where Zajac’s linear first-order model of muscle activation [13] is usually appropriate, and isometric muscle force can be considered a low-pass transform of the rectified EMG. Although first-order muscle activation models have been applied also to muscles of molluscs, crustaceans and insects [2,17–19], they can capture neither the delayed slow rise and rounded peak of a single twitch, nor the non-linear force potentiation observed in the frequency range between 10 and 30 Hz. Our model comparison (Figs 6–9) illustrates that rounded twitch peaks and the frequency-dependent increase in half-maximal rise time typical of invertebrate muscle require the activation model to have higher-order dynamics. On the other hand, frequency-dependent potentiation and change in decay time require a non-linearity. The two models that fulfil these criteria require three times more parameters than first-order linear models, and may require considerably more computation time (Table 3). For this reason, it is worth considering *when* the properties of higher-order non-linear models are of particular relevance, *which* one of the models is preferable, and *what* other aspects require consideration when modelling insect muscle. The following sections address these three questions *when*, *which* and *what*:

### When is the choice of muscle activation dynamics model relevant?

Given the strong, sustained force of a single twitch (Fig 2), and the pronounced non-linear force potentiation for stimulation frequencies between 10 and 30 Hz (Fig 3), one may argue that a muscle activation model should account for these properties whenever the system to be modelled commonly experiences motoneuron frequencies below 30 Hz. On the other hand, even under these conditions, model-dependent differences in isometric force may be small compared to the strong attenuating effects of changes in muscle length and contraction velocity [13]. Thus, in movement sequences where limb kinematics require the use of a wide range of muscle lengths and contraction velocities, variation in muscle force is likely to be governed more by muscle contraction dynamics than by activation dynamics. However, animals often execute the same movement sequence with very similar kinematics, despite marked changes of the mechanical demand. For example, insects can compensate for changes in load without significant changes in kinematics. In this case, length- and velocity-dependent changes in force as described by a muscle *contraction* dynamics model cannot counter the altered mechanical demand. Similarly, passive muscle properties, which have a strong effect on the dynamics of limb movements in insects [1,35–38] cannot account for the compensation of altered load without an associated change in kinematics. In other words, if load compensation occurs without a corresponding change in kinematics, an appropriate change of muscle *activation* is strictly necessary. For example, scratching locusts can compensate for substantial additional loads to the hind leg with essentially no change in joint angle time courses [39]. They control the target-specific movement by appropriate activation of antagonist muscles [32]. In response to a change in load, the associated change in motoneuronal spike number per burst is very small, but this causes a substantial change in co-contraction of antagonist muscles and, as a consequence, a change in net joint torque [1].

In walking or climbing insects, changes in load occur naturally whenever the animal changes its body attitude or encounters a change in inclination of the substrate. For example, during steady-state walking on upward and downward slopes (±45°), stick insects neither change their average speed nor do they show strong changes in leg kinematics. Nevertheless, at the same time, joint torques change drastically with the change in body weight distribution among legs [40]. As for scratching locusts, walking stick insects alter the relative activation of antagonist muscles during the early stance phase, suggesting that they maintain similar kinematics by regulating joint stiffness rather than joint angle or velocity. In both cases, antagonist muscles tend to be activated by alternating bursts of motoneuron activity; but the onset and offset of bursts is not always well defined, and a burst of motoneuron activity in one muscle may be opposed by only a few, or sometimes even a single spike of an antagonist motoneuron. Moreover, owing to the strong and long-lasting time course of a single twitch force, antagonist muscle co-activation can arise even without co-activity of antagonist motoneurons. Thus, even if intermittent spikes between “typical bursts” are rare, the effect of non-linear force potentiation may be substantial because the decay of force after a motoneuron burst may last well into the force build-up cause by an antagonist motoneuron burst. As a consequence, the choice of an appropriate muscle activation model is particularly relevant if the motor behaviour involves co-activation of antagonist muscles, for example for context-dependent regulation of joint stiffness or net torque.

### Which activation dynamics model should be chosen?

The first decision to make when selecting an activation dynamics model should be whether or not a linear model is sufficient for the purpose of modelling. As outlined in the previous section, this decision should depend on whether or not low to medium motoneuron spike rates (1–30 Hz) commonly occur in the movement being modelled. If so, this would call for appropriate consideration of non-linear force potentiation that is most pronounced in this frequency range. On the other hand, if antagonistic motoneurons are consistently firing bursts in alternation and at high frequency (>50 Hz), and temporal separation of antagonistic bursts suggests little or no overlap in the activation time courses of antagonistic muscles, neither non-linear force potentiation nor non-linear effects on force decay would be expected and a linear muscle activation model is likely to be sufficient. Whenever context-dependent modulation of antagonist co-activation occurs (see examples on load compensation above), non-linear force potentiation will be an issue.

Comparing the properties of the two non-linear models, Table 3 shows that the Hatze-Zakotnik model is much more efficient computationally than the non-linear Wilson model. Figs 10 and 11 suggest that modulating a single parameter of the single-twitch model works very well for activation by the slow motoneuron SETi, while revealing considerable mismatch for activation by the fast motoneuron FETi (Table 2; S9 Fig). Apparently, the transition from single twitch to tetanus does not follow the same physiological principles for SETi and FETi activation. Since the non-linear Wilson model does not depend on the modulation of a single twitch, it is possible to find suitable parameter sets for both SETi and FETi activation.

For the slow motoneuron, the Hatze-Zakotnik model has the advantage of being based on a physiologically plausible underlying concept. Twitches are maximally potentiated at a stimulus frequency of 20 Hz (Fig 4), which corresponds approximately to the time from twitch onset to twitch maximum; i.e. maximal potentiation occurs when the following twitch is triggered at the maximum of the preceding twitch. This can also be observed in cat gastrocnemius muscle [40, 41]. Here, potentiation occurs if an additional spike is triggered during the twitch-falling phase, due to reaction kinetics of calcium dynamics, and this leads to increased twitch amplitude and slower force decay. Studies of calcium dynamics in single barnacle muscle fibres show that enhanced release of calcium is the main reason for twitch potentiation [41].

In the Hatze-Zakotnik model, calcium release is included in the second differential equation (Eq 2), in which a single parameter is modulated through a bi-sigmoid Michaelis-Menten type equation (Eq 6). As a formal description of calcium release and removal, it is an intuitive conceptual model of the process underlying the potentiation of twitch force. Frequency-dependent modulation of twitch shape introduces only two additional parameters, and the low complexity makes optimisation of the model parameters to experimental data feasible. This leads to non-redundant solutions, which can be directly compared for different muscles or motoneurons. An extension to the model of muscle force which includes more detail and additional observations can be implemented by including twitch potentiation state as a more complex model parameter. For example, a framework for decomposition of tetanic forces into a series of individual twitches of different sizes was proposed by [42].

As the present study focuses on properties of isometric force time courses under the assumption of no change in muscle fibre length, it is important to note that muscle activation dynamics gets more complicated as soon as muscle is allowed to shorten, or if isometric force time courses are to be compared for different muscle lengths. This is because the force-length dependency of contraction dynamics depends on the level of activation (e.g., [14]). In a recent study, Rockenfeller and Günther discussed a range of activation dynamics models that include length-dependency [16] that transform the normalised calcium concentration γ(t) (Eq 4) into a length-dependent active state q(γ, l), where l denotes the relative fibre length. In their own model and in all of Hatze’s own variants [20,43,44], the nonlinear transform q contains the product of γ and a lever function ϖ(l) (see Table 2 and Eqs 2 and 3 of [16]). Thus, under the assumption of constant fibre length, all of these length-dependent activation dynamics models scale the normalised calcium concentration γ with a constant factor ϖ _fix_. Since none of the nonlinear transforms used in the three model variants discussed here includes length-dependency (Hatze-Zandwijk, Eq 3; Hatze-Zakonik, Eq 5; Wilson non-linear, Eq 8) they can be related to other models by setting ϖ (l) = ϖ _fix_ = 1. Given the non-linear, frequency-dependent force facilitation shown in Fig 11 or S7 Fig, a non-linear transform q(γ) is necessary even without any change in muscle length. Future experiments will need to elucidate the potential interaction of motoneuron firing frequency on the one hand and muscle fibre length on the other.

### What else should be considered when modelling insect muscle?

Both non-linear muscle activation models can account for frequency-dependent, non-linear force potentiation (Fig 10), however, they do not include long-term potentiation of twitches. For example, Brown and colleagues [46] modelled the *sag effect*, i.e., a slow decay in muscle force with long stimulation times, by including increased calcium removal at different stimulus frequencies. The effect of sag on twitches (cf. Fig 4 in [46]) resembles twitch modulation in the Hatze-Zakotnik model, so future work could lead to an incorporation of long-term potentiation into this model.

Another effect not included in current models is long term potentiation of force after a break [26] and the *catch-effect*, i.e., a prolonged increase in force production resulting from as few as one spike in an otherwise constant frequency stimulus sequence (p. 238 in [47]). Catch-like tension is thought to be based on complex calcium dynamics in the muscle [10].

A critical aspect of muscle models in general is that even an optimal set of model parameters can only account for the most typical behaviour in the face of very strong inter-individual variation of muscle properties [48]. Blümel and colleagues showed that for stick insect extensor tibiae muscle contraction dynamics, the use of individually fitted model parameters can halve the error of model estimates [2].

Our own experimental data also suggest that inter-individual variation can result in a considerable range of parameters of activation dynamics (see parameter ranges of the three per-animal fits in Table 2). As a consequence, individually optimised parameter sets lead to different half-maximal rise and decay times (Fig 11), whereas force potentiation appears to be affected less (at least for a given type of model, Fig 10). The fact that the Hatze-Zakotnik and non-linear Wilson models differ substantially more when using the parameters “as published” (Figs 6 to 9; S4 and S5 Figs) than after parameter optimisation to the same data set (Figs 10–12) also indicates that variation among experimental data is substantial. To illustrate this further, we compared the peak forces and half-maximal rise and decay times among three experimental data sets obtained from the same muscle in the same leg of the same insect species, i.e., the hind leg extensor tibiae muscle of the locust *Schistocerca gregaria* ([4,26] and our own data). Frequency-dependent potentiation of peak force was different in our experimental data compared to that of [4,26] for both SETi and FETi (S7 Fig). Our data showed stronger force potentiation at low frequencies. The situation for half-maximal rise time was quite different however: here, the data set of [26] stood out, with up to ten times slower rise and decay of force for low and medium frequencies compared to the data of [4] and our own (S8A Fig). Variation among data sets was smaller in the case of half-maximal rise and decay times during FETi stimulation (S8B Fig).

Physiologically meaningful differences in experimental data both within and between studies may be attributed to a number of factors including inter-individual or genetic strain differences in neuromuscular function, diurnal changes in physiology, the effects of circulating neuromodulators, or direct muscle inhibition. Differences in methodological procedures in different studies could markedly affect modulatory effects in particular. For example, levels of the insect neuromodulator octopamine are elevated following stress [49,50] which may vary among animals and between studies. Octopamine is also released peripherally from Dorsal Unpaired Median (DUM) neurons during particular types of behaviour such as flight or kicking [51,52]. Octopamine modulates both neuromuscular transmission and muscle contractile properties [53]. Moreover, Common Inhibitor motoneurons (for review see [54]) release γ-aminobutyric acid (GABA) onto insect skeletal muscles, which influences muscle contraction and relaxation dynamics [55–57]. In the locust, Common Inhibitor activation reduces extensor muscle relaxation times [58] and may reduce the force generated by excitatory motor spikes, thus facilitating fast cyclical leg movements [56,57,59]. For pharmacological induction of muscle relaxation related to inhibitory innervation see [60].

In summary, much of the difference between the two published non-linear activation dynamics models is due to considerable differences between the experimental data sets, but this inter-individual variation is an important aspect of muscle physiology. In the face of this inter-individual variation, a test for generality of any computational model involving muscle properties will require systematic variation of model parameters within the documented parameter ranges. Our muscle activation toolbox for Matlab [61] and the corresponding experimental data set [5] will facilitate the comprehensive and computationally efficient use of different activation dynamics models, and will also help us to learn more about variation of muscle properties in different preparations, types of muscle, and species.

## Materials and methods

### Muscle force recordings

Experiments were carried out on adult male and female *Schistocerca gregaria*, from crowded colonies at the Department of Zoology, University of Cambridge, UK or Department of Biology, University of Leicester, UK. Locusts were fixed in modelling clay, ventral side uppermost. The right hind leg was immobilised with dental cement (Protemp, ESPE) with the femur at right angles to the body, and the femoro-tibial angle initially set at 140° or 90°, for SETi and FETi experiments respectively to facilitate the subsequent attachment of the extensor muscle to the force transducer. For SETi experiments, a window was cut in the distal end of femur, and the accessory flexor muscle, overlying trachea and air sacs were removed. The end of the apodeme of the extensor muscle was grasped with a pair of forceps attached to a force transducer (see below) and the apodeme was cut distal to the forceps. Stimulation of FETi causes high extensor muscle forces that can fracture the extensor muscle apodeme at the point where it is grasped by forceps. To avoid this in FETi experiments, the proximal tibia was first braced in an aluminium sleeve which was then tightly attached to the force transducer with suture thread, such that the attachment point was aligned with the axis of pull of the extensor muscle. The distal femoral cuticle of the femoro-tibial joint was dissected away, so that the extensor muscle remained attached solely to the transducer through its natural point of attachment to the dorsal proximal tibia. For both SETi and FETi experiments, the extensor muscle length was adjusted to correspond to 90° femur-tibia angle before stimulation.

### Stimulation protocol

A window was cut into the ventral thoracic cuticle, and overlying air sacs were removed. Contractions in the extensor tibiae muscle were elicited by stimulation of Nerve 3b (N3b) or Nerve 5 (N5) in the thorax, for SETi and FETi respectively, through a pair of 50 μm silver hook electrodes placed under the nerve and insulated with petroleum jelly. Stimulation strength was set just above the threshold for eliciting a single twitch reliably. The axon of common inhibitor motoneuron CI_1_ runs in the same nerve as that of SETi, but it was not activated in these experiments. This was confirmed by making intracellular recordings from slow extensor tibiae muscle fibres during N3b stimulation. Such recordings revealed excitatory junctional potentials (from SETi) but no inhibitory potentials [62].

Stimuli were generated using a Master 8 stimulator (AMPI, Jerusalem, Israel). Series of pulses at various frequencies were delivered for 10 s or 1 s, for SETi and FETi respectively, with a 60 s gap between each stimulus train to allow the muscle to recover. FETi stimulus trains were restricted to 1 s to prevent damage to the apodeme insertion point. Muscle forces were measured using an isometric force transducer (Model 305C, Aurora Scientific, Canada), digitised at 5 kHz using a micro1401 interface and Spike 2 software (both Cambridge Electronic Design, Cambridge, UK).

### Model implementation

To focus on the time course of muscle activation, measured forces were normalised to the interval [0,1], where 1 corresponds to maximum measured force per animal. They were not filtered. In the model, the stimulus protocol was shifted by a fixed delay (e.g. 10 ms) to account for the time taken by neural signals to be conducted to the muscle from the point of nerve stimulation.

Either constrained Levenberg-Marquardt or trust-region-reflective optimisation algorithms, implemented in Matlab (Mathworks Inc, Natick, USA), were used to fit the model parameters. These algorithms minimised a least squares error function that captured the distance between the measured and stimulated forces. The fitting procedure was repeatedly initialised with randomly distributed values to avoid local minima. Both algorithms were applied to the same experimental data set, and the one with the better performance (lower error) used for further analysis. This is mentioned in the data structures of the optimised data provided with the supplementary MATLAB IMADSim toolbox [61]. In the following sections, we introduce the five muscle activation models compared in this study.

### Hatze-Zakotnik model

Hatze [20, 44] proposed a second-order model comprising a pair of coupled second-order inhomogeneous differential equations, to capture two stages of signal processing (Eqs 16 and 17 in [20]; Eqs 3.21 and 3.22 with ρ*(ξ) = 1 in [44]). The first stage describes the transformation of neural activity α(t) at the motor endplate to membrane potential β(t) in the T-tubular system of a muscle. α(t) is zero except for 1 ms periods in which each motoneuron spike is modelled as a half sine wave, idealising the depolarised portion of an action potential (S1 Fig). When entering the T-tubular system, the action potential α(t) is transformed to signal β(t) according to the differential Eq 1:

$$\frac{\partial^{2}\beta }{\partial t^{2}}+\theta_{1}\frac{\partial \beta }{\partial t}+\theta_{2}\beta =\alpha \left(t\right)$$ (Eq 1)

The second stage of the model transforms β(t) into the intracellular free ionic calcium concentration [Ca^2+^]_i_ and therefore models calcium release and re-uptake by the sarcoplasmic reticulum. Differential Eq 2 determines the calcium concentration γ(t):

$$\frac{\partial^{2}\gamma }{\partial t^{2}}+\theta_{3}\frac{\partial \gamma }{\partial t}+\theta_{4}\gamma =\beta \left(t\right)$$ (Eq 2)

Both processes can be viewed as over-critically damped, second-order systems, each with a different parameter set [20]. See Part I of the Supplementary Appendix S10 for a numerical solution for γ(t).

The relationship between the concentration of released calcium and the active state of the muscle is typically described by a sigmoid function as measured in [63] and [64]. Hatze [20] proposed that the active state also depended on muscle fibre length (his Eq 14), and varied this dependency in different versions (for a detailed treatise of this length dependence and its significance, see [16]). Given the lack of experimental data with systematic co-variation of both motoneuron frequency and muscle fibre length, the present study focuses on variants of the Hatze model that assume muscle fibre length to remain constant. For example, van Zandwijk and colleagues [45] expanded the Hatze model by including a sigmoid relationship between the calcium concentration, i.e., γ(t), and the force-producing active state:

$$q\left(\gamma \right)=\frac{1}{1+\exp\left[A\left(\log\gamma -\log\gamma_{0}\right)\right]},$$ (Eq 3)

where A and γ_0_ describe the slope and offset of the sigmoid, respectively. Owing to an unsatisfactory fit of the Hatze-van-Zandwijk model to isometric force measurements in insect muscle (S2 Fig), Zakotnik [65] replaced van Zandwijk’s sigmoid (Eq 3) with a frequency-dependent potentiation factor c(f) (hence the name Hatze-Zakotnik model). Reordering of Eq 2 shows that the calcium concentration γ is divided by the parameter value θ_4_:

$$\gamma \left(t\right)=\frac{\beta (t)-\frac{\partial^{2}\gamma }{\partial t^{2}}-\theta_{3}\frac{\partial \gamma }{\partial t}}{\theta_{4}}$$ (Eq 4)

Therefore, if value θ_4_ is decreased, the twitch force γ increases and has a slower decay. In contrast, parameter θ_3_ controls the twitch shape such that tetanic force does not change (Fig 1). To model force potentiation in the Hatze-Zakotnik model, θ_4_ is multiplied by a factor c(f) that depends on the instantaneous motor spike frequency f:

$$\gamma \left(t\right)=\frac{\beta (t)-\frac{\partial^{2}\gamma }{\partial t^{2}}-\theta_{3}\frac{\partial \gamma }{\partial t}}{c\left(f\right)\cdot \theta_{4}}$$ (Eq 5)

Assuming that there is no potentiation for a single twitch (f = 1, c(1) = 1), values for c(f) can be determined by optimising parameter θ_4_ for each stimulation frequency independently and dividing this value by the measured θ_4_ at single twitch stimulation. Note that, for this procedure, the other three parameters, θ_1_ to θ_3_, remain fixed.

Because calcium kinetics are thought to be the main reason for force potentiation, a Michaelis-Menten-type equation is used for c(f). It is related to a [Ca^2+^]_i_ reaction process that potentiates a twitch, and a calcium pump that reduces both [Ca^2+^]_i_ and twitch size. The value for c(t) depending on the time since the previous stimulus t = (1/f) is determined as:

$$c\left(t\right)=\frac{t^{2}}{K_{1}+t^{2}}-\frac{t^{2}}{K_{2}+t^{2}}+1$$ (Eq 6)

Eq 6 relies on two parameters K_1_ and K_2_, for which we assume that K_1_ ≥ K_2_ and K_1_, K_2_ ≥ 0. Therefore, it is constrained to the interval [0, 1] and converges to 1 for t → ∞. The first term can be related to the force-producing reaction; i.e., larger values of K_1_ produce an elevated and prolonged twitch force. The second term can be related to the removal of calcium, i.e., larger values of K_2_ reduce twitch potentiation.

The parameters, K1 and K2 were also optimized by using either constrained Levenberg-Marquardt or trust-region-reflective algorithms, to fit the c(f) curve. It should be noted that if the number of data points (here, the number of different stimulation frequencies) is small, the fitted curve could deviate slightly more at certain frequency points (see the IMADSim optimisation example given in the supplementary toolbox [61]). Ultimately, this will affect the performance of the full six-parameter Hatze-Zakotnik model. For example, in Fig 10A and 10C, a larger deviation can be seen in FETi force prediction (where 5 frequencies were used for optimisation) compared to that of SETi (with 10 different frequencies).

### Non-linear Wilson model

We compare the Hatze-Zakotnik model with another non-linear second order model that was proposed by Wilson and colleagues [4]. The authors adapted and simplified the model of Ding [66] to obtain second order dynamics, and used it to describe the isometric response of locust skeletal muscle to SETi stimulation [4].

The Wilson model gives the muscle force *F(t)* and takes a pulse train *u(t)* as an input. The model equations are:

$$\frac{dC_{N}(t)}{dt}+\frac{C_{N}(t)}{\tau_{c}}=u(t)$$ (Eq 7)

$$x(t)=\frac{C_{N}{\left(t\right)}^{m}}{C_{N}{\left(t\right)}^{m}+k^{m}}$$ (Eq 8)

$$\frac{dF(t)}{dt}+\frac{F(t)}{\tau_{1}+\tau_{2}x(t)}=A\cdot x(t)$$ (Eq 9),

where

$$u(t)=\sum_{i=1}^{n}\delta (t-t_{i})$$ (Eq 10)

Here, *n* defines the number of input pulses, *t* the time and *t*_*i*_ the time at which the i^th^ pulse occurs. This accounts for the assumption that the input pulses can be approximated as impulses. Therefore, the motoneuron spike is modelled as a square pulse or half-sine wave of width 1 ms, scaled to have an area of 1 under the pulse. Like the Hatze-Zakotnik model, the non-linear Wilson model has six parameters, but it consists of first-order differential equations. The variable *x(t)* is an intermediate stage in the model and represents a non-linear saturation. The parameters *m* and *k* define the shape of this non-linearity. A measure of [Ca^2+^]_i_ is represented by the variable *C*_*N*_. The parameters τ_c_, τ_1_ and τ_2_ are time constants, and *A* is a gain [4]. Note that Eqs 7 and 8 are very similar to a computationally efficient approximation of the original model as formulated by Hatze (see Eqs 3.27 and 3.29 to 3.31 in [44], where the normalised calcium concentration C_N_(t) is the equivalent to γ(t) and the nonlinear transform x(t) is equivalent to q(γ(t)) without length-dependency).

The parameter set for each animal (i.e. muscle) was estimated by minimising the least squares error between the measured force and the model output for the entire set of trials per animal, i.e., for single twitch and all stimulus frequencies. As described by Wilson et al. [4], the fitting procedure was initialised repeatedly seven times, with random parameter values drawn from a normal distribution. The optimisation was done using the MATLAB function *lsqnonlin* and the trust-region-reflective algorithm was used to find the best fit to the data. The system of differential equations was solved using a fixed step (0.0002 s or 5 kHz sampling rate), fourth-order Runge-Kutta method.

### Zajac model

In a reference work on muscle modelling, Zajac [13] proposed a linear first-order model for activation dynamics. The envelopes of the rectified electromyogram (EMG) and of the low-pass filtered, rectified EMG, can be related to the neural-excitation input signal *u(t)* and the state variable associated with muscle activation *a(t)*, respectively. It is a two-parameter model described by the following bilinear differential equation:

$$\frac{da(t)}{dt}+\left[\frac{1}{\tau_{act}}(\beta +\left[1-\beta \right]u(t))\right]a(t)=\frac{1}{\tau_{act}}u(t)$$ (Eq 11)

$$0<\beta =\frac{\tau_{act}}{\tau_{deact}}<1$$ (Eq 12)

where 1/τ_act_ is the higher rate constant (when *u(t)* = 1) and β is the ratio of that over the lower rate constant, 1/ τ_deact_, when *u(t)* = 0 (relaxation). In other words, the model assumes that the build-up of activation of a fully excited muscle is faster than relaxation after termination of activation. In the case of the Zajac model, we use an iterative method for solving the differential equation numerically, as described in part II of the S1 Text.

### Blümel model

Blümel and colleagues [2] proposed a simple model of activation dynamics for the stick insect mesothoracic extensor tibiae muscle. First-order low-pass filtering reproduces many aspects of isometric contractions in this muscle [67], so the activation dynamics are described by a single-pole, first-order low pass filter. The standard recursion equation for such a filter was used to obtain the following two-parameter model:

a[n]=(1−filter)⋅(scaling⋅u[n])+filter⋅a[n−1], (Eq 13)

where *filter* sets the decay amplitude per time step and *scaling* is a factor that multiplies the input by a constant. *u[n]*and *a[n]* correspond to neural excitation and muscle activation, respectively. The time constant, t_const_, of the filter depends on the time step duration and is related to the parameter filter according the following equation:

$$t_{const}=-\frac{\Delta t}{\ln(filter)},$$ (Eq 14)

where Δt is the time step duration. In our case, Δt was 0.0002 s in all simulations.

### Linear Wilson model

Wilson and colleagues [3], found that a third-order model was of optimal order for fitting isometric force responses over a range of input pulse frequencies. Their linear third-order model of activation dynamics is characterised as:

$$\theta_{3}\frac{d^{3}a(t)}{dt^{3}}+\theta_{2}\frac{d^{2}a(t)}{dt^{2}}+\theta_{1}\frac{da(t)}{dt}+a(t)=\theta_{0}u(t),$$ (Eq 15)

where *a(t)* is the muscle force and *u(t)* is the input pulse train. The model has four parameters, θ_1_ *to* θ_4_. Here, we converted the third-order differential Eq 15 into a system of three first-order differential equations and solved it by using a fixed-step (0.0002 s), fourth-order Runge-Kutta method.

### MATLAB Toolbox and sample data

For quantitative comparison of the five models described above, with particular focus on their responses to constant frequency pulse trains, we developed a MATLAB toolbox IMADSim, or “Insect Muscle Activation Dynamics Simulation”. The toolbox consists of Matlab routines for simulating the models for arbitrary pulse trains and arbitrary parameter combinations. It was created in Matlab2009b (7.9.0) and comes with an additional installer file for Matlab versions above 2014 [61].

A graphical user interface (GUI) is provided so that a user can select a muscle activation model, adjust model or simulation parameters easily, and visualise the output (S3 Fig). The GUI permits the selection of one of two motoneuron types (SETi or FETi), spike shapes (half-sinusoidal or square) and spike generators (either constant frequency or Poisson). Arbitrary spike trains may be loaded from a file (in .mat or .txt format, comprising spike times). For constant spike frequencies, single or multiple spike frequencies can be set along with a relaxation time. There are three displays showing: (i) the time course of isometric force generation, (ii) the corresponding spike train, and (iii) the model equations. Users can save all graphs generated, as well as the corresponding data (Matlab file formats .fig or .mat).

The toolbox also includes parameter optimisation routines for the two non-linear models, i.e. the Hatze-Zakotnik and non-linear Wilson model, which are based on the Matlab LSQNONLIN solver for non-linear least squares problems. When calling the functions, several arguments can be set to customise the optimisation. If no arguments are given, default values are used. The toolbox assumes that experimental data are sampled at 5 kHz.

In addition to the toolbox, we provide experimental data from six adult female locusts (*Schistocerca gregaria*), comprising isometric contraction force time courses of the metathoracic extensor tibiae muscle for spike trains with different frequencies (http://doi.org/10.4119/unibi/2937068, [5]). In three animals, the slow extensor tibiae motoneuron (SETi) was stimulated. For another three animals, the fast extensor tibiae motoneuron (FETi) was stimulated.

For more details about the toolbox, functional routines, and examples, the reader is referred to the documentation file “IMADSim_Documentation.html” in the Supplementary Material [61].

## Supporting information

S1 Fig Motor spike approximation. Stimulus shape recorded in experiments (solid line) and used in the model (dashed line). The model stimulus is a half sine wave of length 1 ms and approximates well the depolarising phase of the signal.
(TIF)

S2 Fig The Hatze-van-Zandwijk model, with non-linear scaling of twitch force. Blue lines show isometric force measurements for locust extensor muscle at different SETi stimulus frequencies are shown in the left panel (same data as in Fig 3). Forces were normalised to maximum force at a stimulation frequency of 50 Hz. Note that the tetanic force level difference between e.g. 10 and 20 Hz is larger than the difference between e.g. 40 and 50 Hz, which indicates a non-linear summation of single twitch forces. Black lines show simulated forces using the Hatze-van-Zandwijk model (Eq 1 to 3). The four parameters of Hatze’s original activation dynamics model were optimised to the single twitch of A. Van Zandwijk proposed a sigmoid scaling function (Eq 3, see inset on the right) to model the relationship between the calcium concentration and the force-producing active state. Comparison with the experimental data (blue lines) shows that the model fit is poor. For example, the tetanus fuses only at frequencies above 25 Hz and the rise time at higher stimulation frequencies is too short.
(TIF)

S3 Fig Graphical User Interface (GUI) of the insect muscle activation dynamics simulation toolbox for Matlab. The main panel of the GUI permits the selection of one of five published muscle activation models (two non-linear and three linear). The user can manually alter all corresponding model parameters, select one of two motor neuron types (SETi and FETi), toggle between two spike shapes (sine and square) and select one of two methods for spike generation. Experimental spike time series may be loaded from an external Matlab or text file that contains a list of individual spike times. For constant frequency stimulation, one or more spike frequencies may be set (1, 5 and 20 Hz, in the example shown). Post-stimulation relaxation time may also be set. Three displays show: (i) the time course of isometric force generation, (ii) the corresponding spike train, and (iii) the model equations. Finally, “Hold on”, “Run” and “Reset all” buttons are used to keep the multiple time courses on the display, run the simulation, or reset to default settings, respectively. Generated graphs and the corresponding data can be saved to Matlab figure (*.fig) and data (*.mat) files by selecting options in the toolbar.
(TIF)

S4 Fig Comparison of the Hatze-Zakotnik and Wilson non-linear models with muscle forces induced by fast motor neuron (FETi) stimulation. Time courses of isometric force contractions for different trains of constant frequency stimulation are shown for the two non-linear models with parameters ‘as published’ according to Hatze-Zakotnik (**A**) and Wilson (**B**). Note that model output was normalised to maximum force of the single-twitch. This was set to 0.1. Same figure details as in the top row of Fig 6, except that here the fast motor neuron (FETi) was simulated.
(TIF)

S5 Fig Model response to random activity of a fast motoneuron. Same figure details as in Fig 7, except that here the fast motor neuron (FETi) is stimulated.
(TIF)

S6 Fig Force decay predicted by the Hatze-Zakotnik model. Time courses of force decay after 10 s of constant frequency SETi stimulation and 1 s of constant frequency FETi stimulation were superimposed for the model (black) and experimental data (coloured). A: SETi, blue. B: FETi, red. The onset of the last stimulus spike is set at t = 0. Numbers at the start of decay indicate stimulation frequencies in Hz. Although the shape of the force signal is similar in model and experiment for SETi stimulation, the experimentally measured decay after stimulation with 20 or 40 Hz lags the onset of the modelled decay. The same is true for FETi stimulation at 50 Hz. For stimulation frequencies of 10 and 20 Hz, the experimentally measured FETi time courses show much slower decay than those computed by the model.
(TIF)

S7 Fig Comparison of peak forces in different experimental data sets. Three different data sets are compared for SETi (**A**) and FETi (**B**) stimulation at different frequencies. The data sets comprise our own experimental data (black; 3 animals, per motoneuron), data published by [26] (SETi: cyan; his Fig 3F for single twitch and Fig 20 for inner and outer muscle fibre bundles; FETi: magenta; his Fig 3B for single twitch and Fig 15B for outer muscle fibre bundle), and data published by [4] (SETi: dashed blue; their Fig 2E; FETi: dashed red; their Fig 2F). Forces were normalised to the peak force at stimulation frequency 50 Hz separately for SETi and FETi.
(TIF)

S8 Fig Comparison of half-maximal rise and decay times in different experimental data sets. Same three experimental data sets as used in S7 Fig.
(TIF)

S9 Fig Comparison of non-linear models optimised to the same experimental data. Model fits (black) of the Hatze-Zakotnik model (top) and non-linear Wilson model (bottom) to experimental data sets for SETi (blue) and FETi (red) stimulation. Plots for animal D (SETi) and animal 4 (FETi) show the same data in Fig 10 except that forces were not normalised to maximum force at 50 Hz stimulation frequency. For model parameter sets used see Table 2. Constant stimulating frequencies used were: 1, 7, 10, 12.5, 15, 20, 25, 30, 40 and 50 Hz for SETi, and 1, 10, 20, 30 and 50 Hz for FETi.
(TIF)

S1 Text I. An implementation of Ordinary Differential Equations (ODE) for the Hatze-Zakotnik model. II. Recursion equation to solve the ODE for the Zajac model.
(PDF)
