# Supplementary figures and images for: Evaluation of linear and non-linear activation dynamics models for insect muscle
Source: PLoS Comput Biol. 2019 Oct 14;15(10):e1007437. doi: 10.1371/journal.pcbi.1007437 (PMC6812852; doi:10.1371/journal.pcbi.1007437)

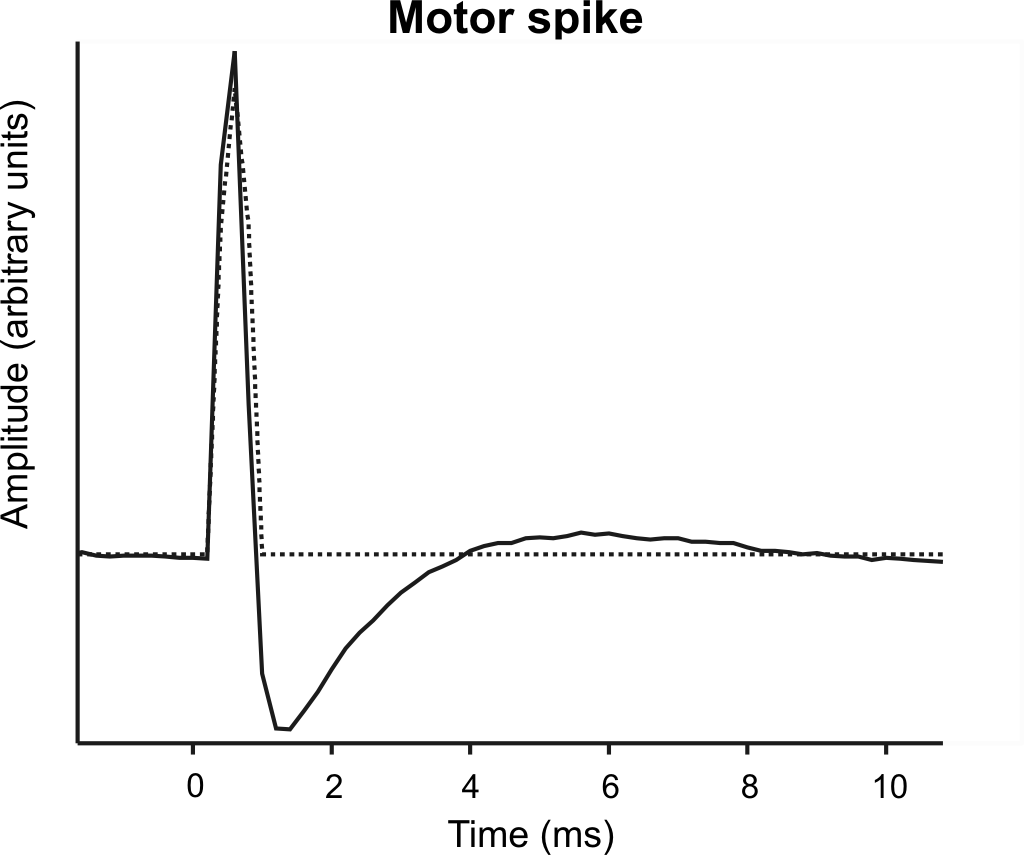

Supplement: S1 Fig — Stimulus shape recorded in experiments (solid line) and used in the model (dashed line). The model stimulus is a half sine wave of length 1 ms and approximates well the depolarising phase of the signal. (TIF) [file pcbi.1007437.s001.tif]

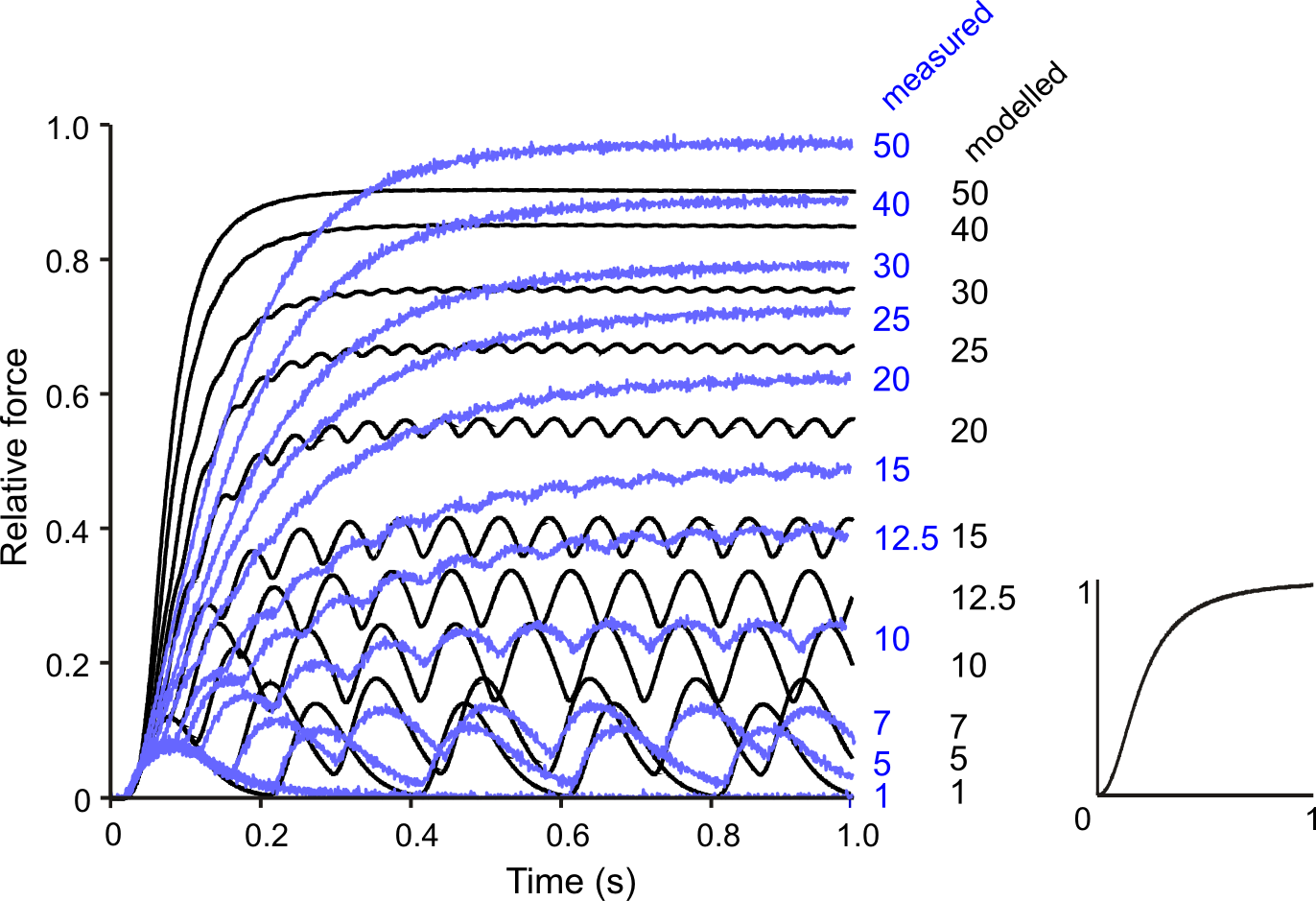

Supplement: S2 Fig — Blue lines show isometric force measurements for locust extensor muscle at different SETi stimulus frequencies are shown in the left panel (same data as in Fig 3). Forces were normalised to maximum force at a stimulation frequency of 50 Hz. Note that the tetanic force level difference between e.g. 10 and 20 Hz is larger than the difference between e.g. 40 and 50 Hz, which indicates a non-linear summation of single twitch forces. Black lines show simulated forces using the Hatze-van-Zandwijk model (Eq 1 to 3). The four parameters of Hatze’s original activation dynamics model were optimised to the single twitch of A. Van Zandwijk proposed a sigmoid scaling function (Eq 3, see inset on the right) to model the relationship between the calcium concentration and the force-producing active state. Comparison with the experimental data (blue lines) shows that the model fit is poor. For example, the tetanus fuses only at frequencies above 25 Hz and the rise time at higher stimulation frequencies is too short. (TIF) [file pcbi.1007437.s002.tif]

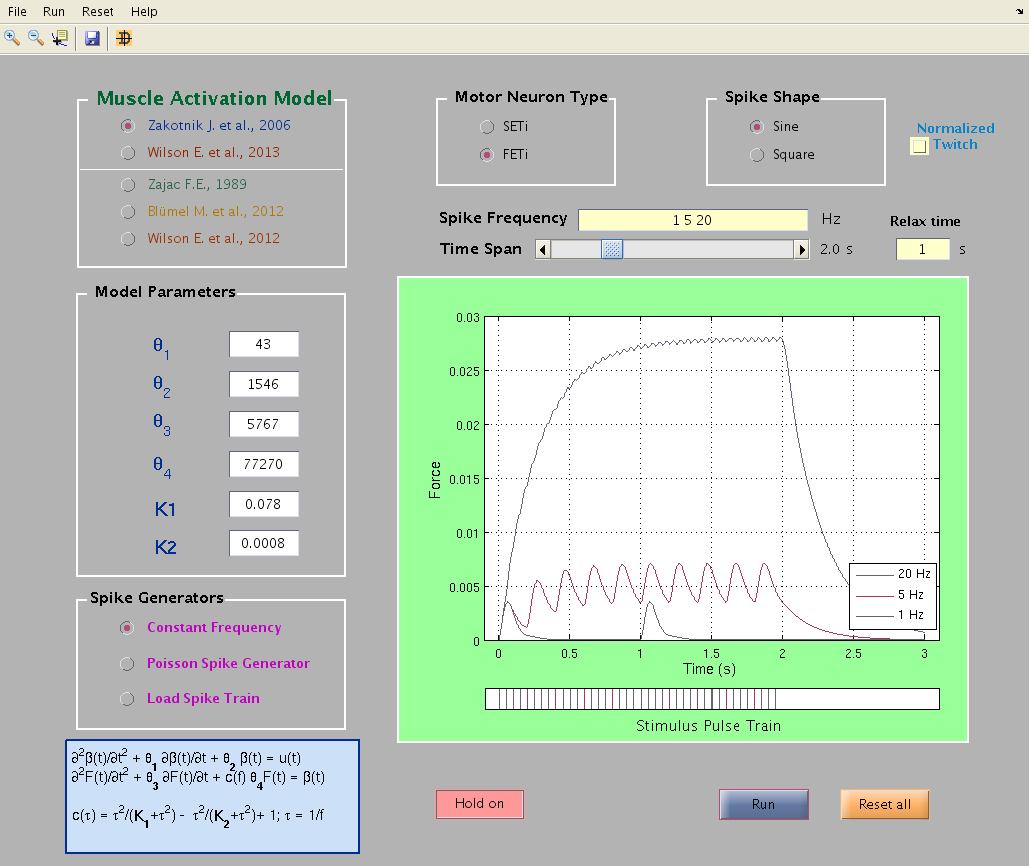

Supplement: S3 Fig — The main panel of the GUI permits the selection of one of five published muscle activation models (two non-linear and three linear). The user can manually alter all corresponding model parameters, select one of two motor neuron types (SETi and FETi), toggle between two spike shapes (sine and square) and select one of two methods for spike generation. Experimental spike time series may be loaded from an external Matlab or text file that contains a list of individual spike times. For constant frequency stimulation, one or more spike frequencies may be set (1, 5 and 20 Hz, in the example shown). Post-stimulation relaxation time may also be set. Three displays show: (i) the time course of isometric force generation, (ii) the corresponding spike train, and (iii) the model equations. Finally, “Hold on”, “Run” and “Reset all” buttons are used to keep the multiple time courses on the display, run the simulation, or reset to default settings, respectively. Generated graphs and the corresponding data can be saved to Matlab figure (*.fig) and data (*.mat) files by selecting options in the toolbar. (TIF) [file pcbi.1007437.s003.tif]

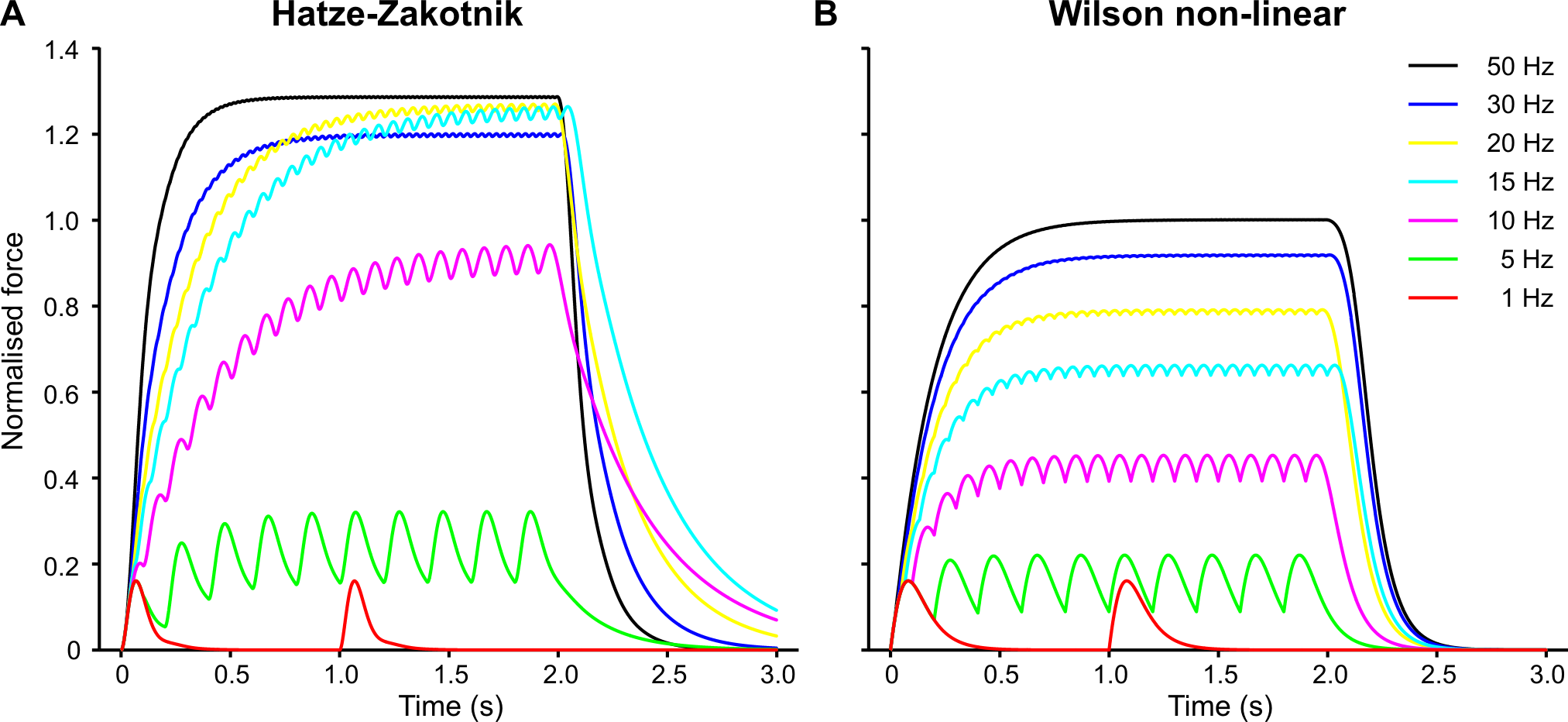

Supplement: S4 Fig — Time courses of isometric force contractions for different trains of constant frequency stimulation are shown for the two non-linear models with parameters ‘as published’ according to Hatze-Zakotnik (A) and Wilson (B). Note that model output was normalised to maximum force of the single-twitch. This was set to 0.1. Same figure details as in the top row of Fig 6, except that here the fast motor neuron (FETi) was simulated. (TIF) [file pcbi.1007437.s004.tif]

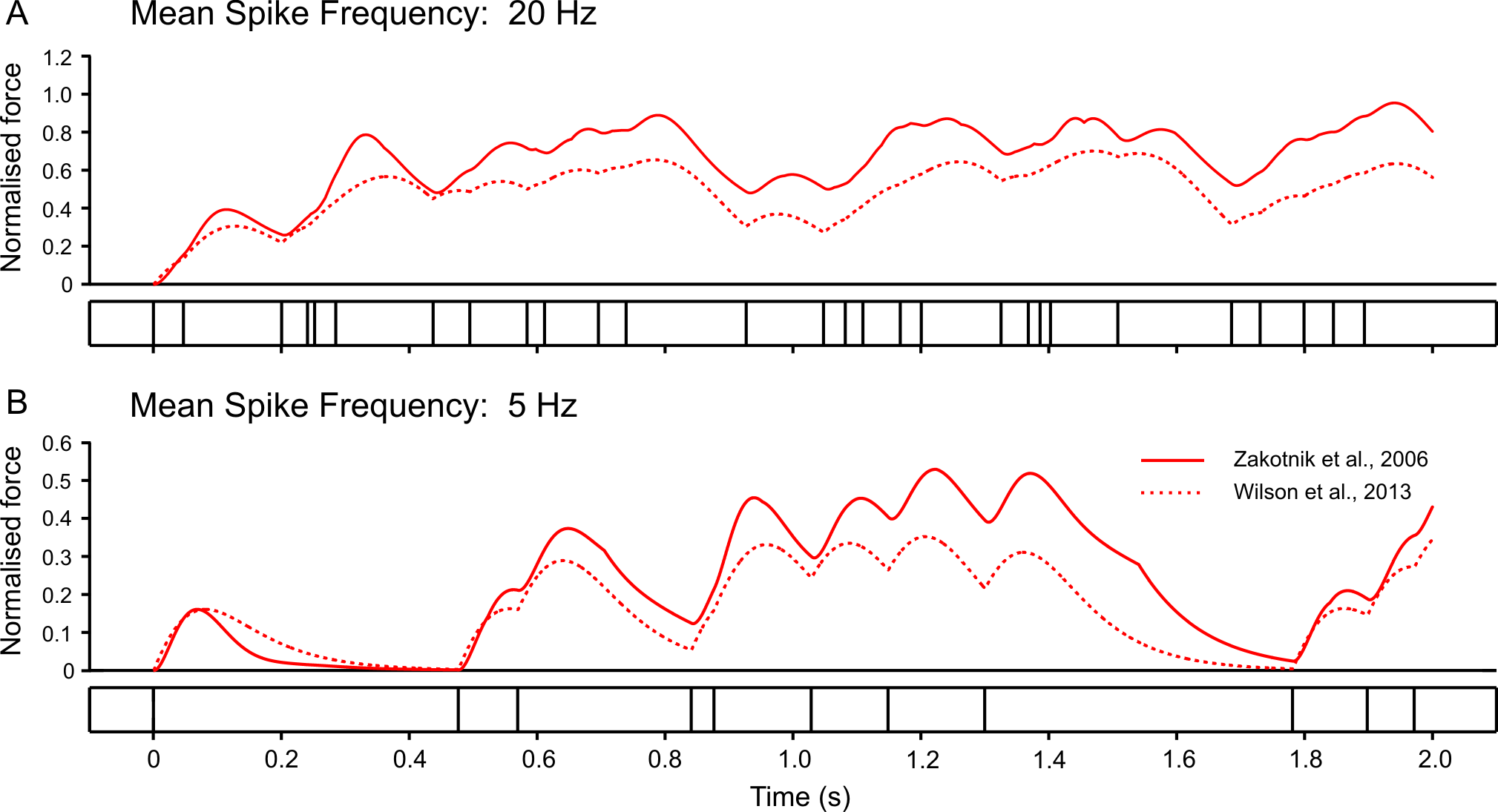

Supplement: S5 Fig — Same figure details as in Fig 7, except that here the fast motor neuron (FETi) is stimulated. (TIF) [file pcbi.1007437.s005.tif]

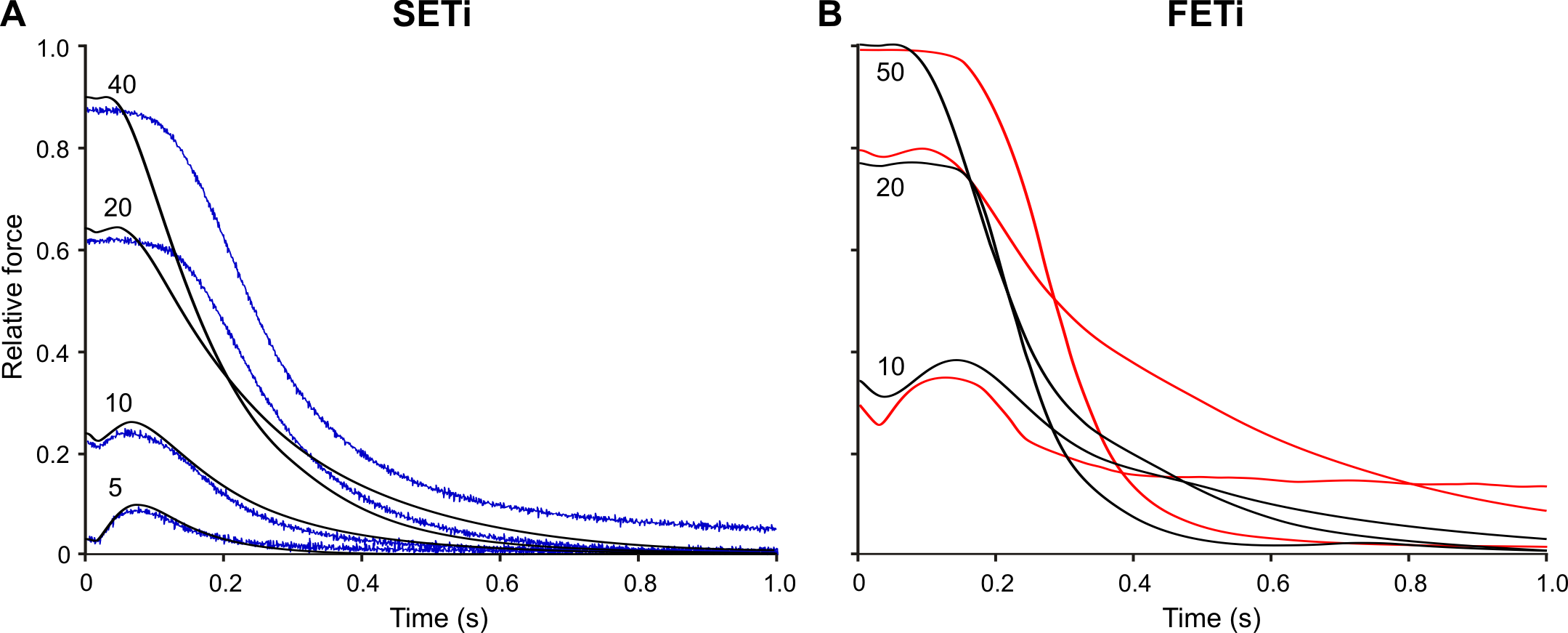

Supplement: S6 Fig — Time courses of force decay after 10 s of constant frequency SETi stimulation and 1 s of constant frequency FETi stimulation were superimposed for the model (black) and experimental data (coloured). A: SETi, blue. B: FETi, red. The onset of the last stimulus spike is set at t = 0. Numbers at the start of decay indicate stimulation frequencies in Hz. Although the shape of the force signal is similar in model and experiment for SETi stimulation, the experimentally measured decay after stimulation with 20 or 40 Hz lags the onset of the modelled decay. The same is true for FETi stimulation at 50 Hz. For stimulation frequencies of 10 and 20 Hz, the experimentally measured FETi time courses show much slower decay than those computed by the model. (TIF) [file pcbi.1007437.s006.tif]

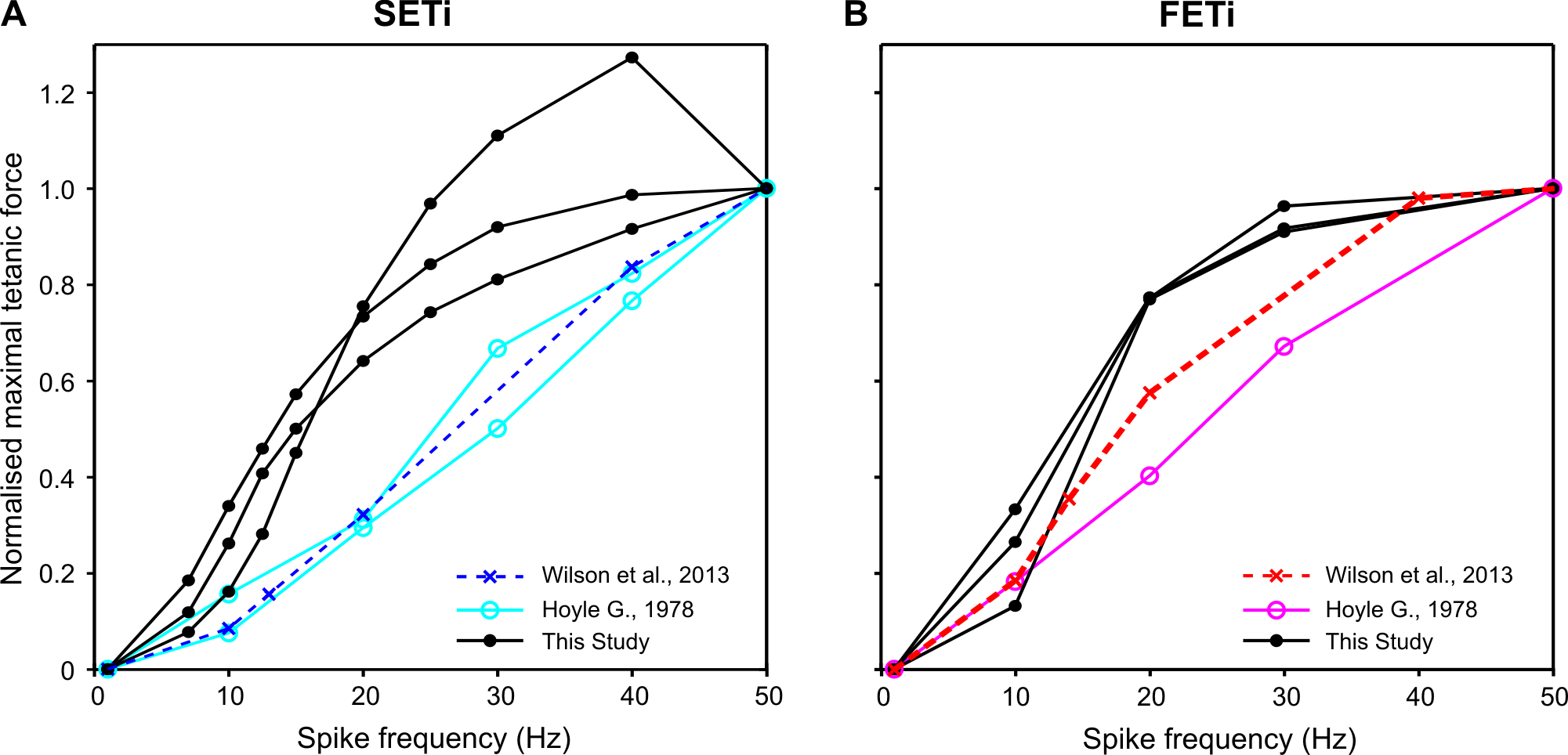

Supplement: S7 Fig — Three different data sets are compared for SETi (A) and FETi (B) stimulation at different frequencies. The data sets comprise our own experimental data (black; 3 animals, per motoneuron), data published by [26] (SETi: cyan; his Fig 3F for single twitch and Fig 20 for inner and outer muscle fibre bundles; FETi: magenta; his Fig 3B for single twitch and Fig 15B for outer muscle fibre bundle), and data published by [4] (SETi: dashed blue; their Fig 2E; FETi: dashed red; their Fig 2F). Forces were normalised to the peak force at stimulation frequency 50 Hz separately for SETi and FETi. (TIF) [file pcbi.1007437.s007.tif]

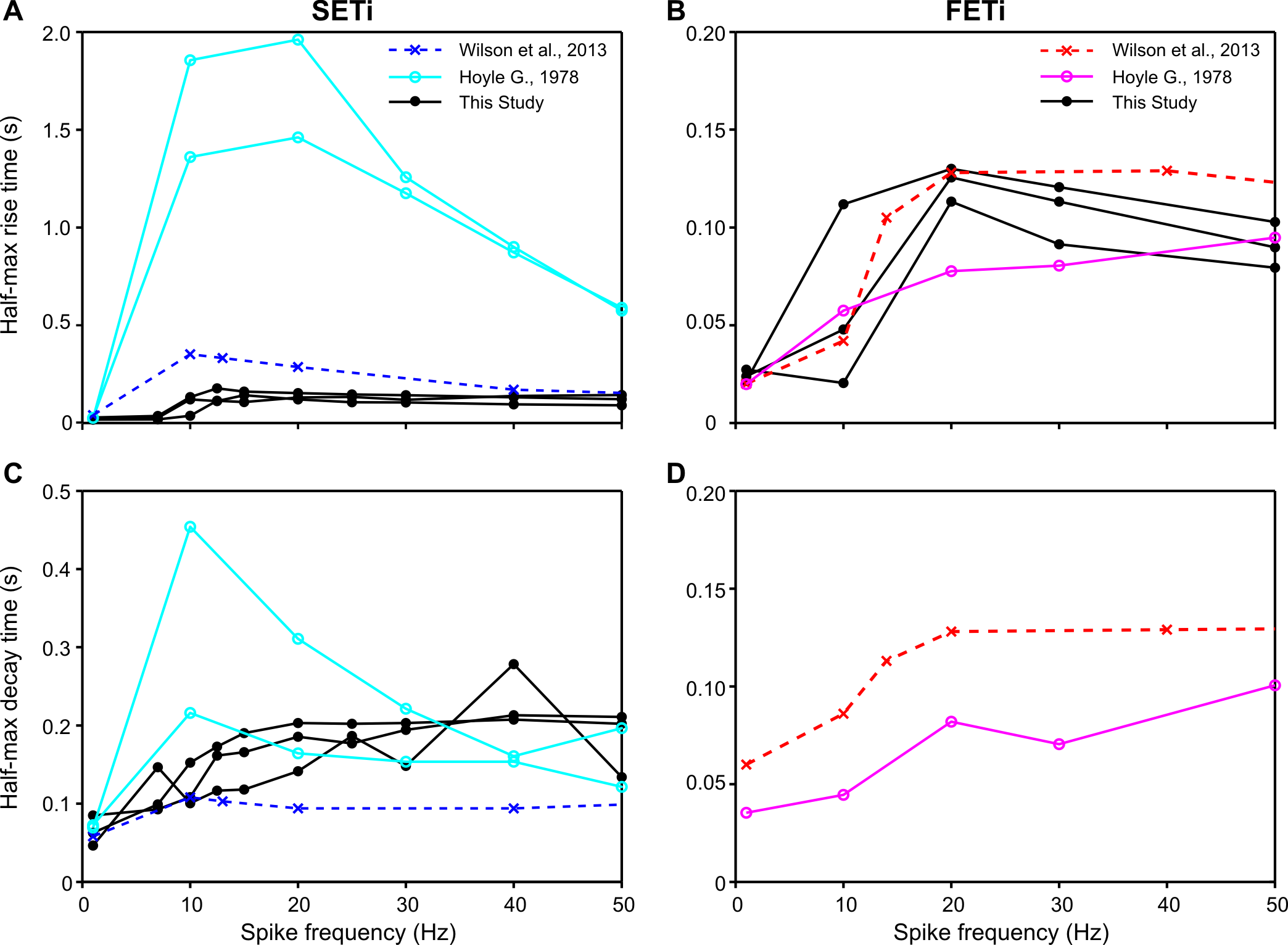

Supplement: S8 Fig — Same three experimental data sets as used in S7 Fig. (TIF) [file pcbi.1007437.s008.tif]

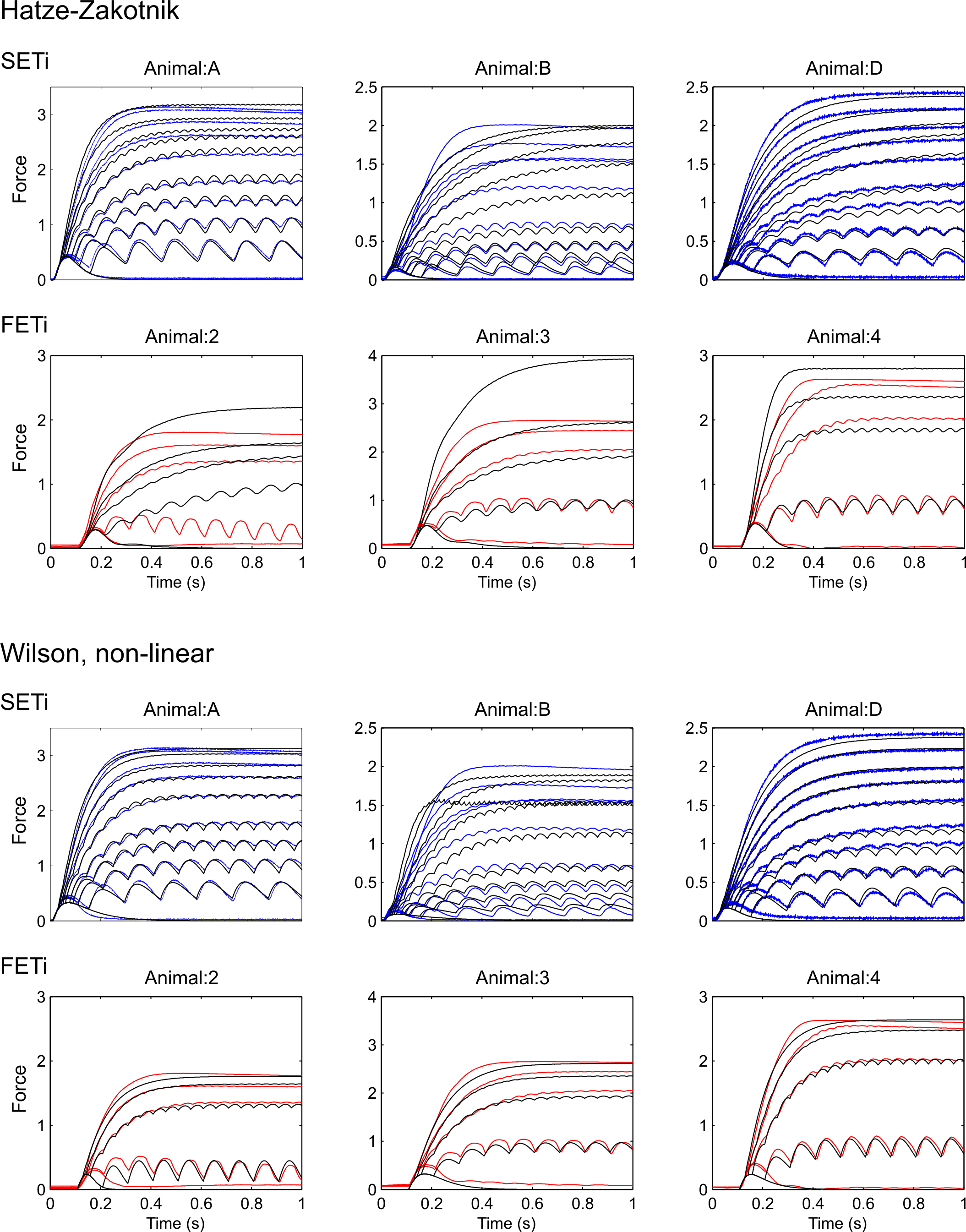

Supplement: S9 Fig — Model fits (black) of the Hatze-Zakotnik model (top) and non-linear Wilson model (bottom) to experimental data sets for SETi (blue) and FETi (red) stimulation. Plots for animal D (SETi) and animal 4 (FETi) show the same data in Fig 10 except that forces were not normalised to maximum force at 50 Hz stimulation frequency. For model parameter sets used see Table 2. Constant stimulating frequencies used were: 1, 7, 10, 12.5, 15, 20, 25, 30, 40 and 50 Hz for SETi, and 1, 10, 20, 30 and 50 Hz for FETi. (TIF) [file pcbi.1007437.s009.tif]
