# Supplementary material for: Evaluation of linear and non-linear activation dynamics models for insect muscle
Source: PLoS Comput Biol. 2019 Oct 14;15(10):e1007437. doi: 10.1371/journal.pcbi.1007437 (PMC6812852; doi:10.1371/journal.pcbi.1007437)
Supplement: S1 Text — I. An implementation of Ordinary Differential Equations (ODE) for the Hatze-Zakotnik model. II. Recursion equation to solve the ODE for the Zajac model. (PDF) [file pcbi.1007437.s010.pdf]

Supplementary Appendix

I. An implementation of Ordinary Differential Equations for the Hatze-Zakotnik model

The evaluation of Eq. 1 and 2, which determine the muscle response β from an experimental spike train input α, is computationally intensive. It is therefore important to implement the calculation efficiently. Note that α(t) are the measured values of the spike-triggering signal and are therefore not given as a function.

Both [1] and [2] used numerical methods applying a differential equation solver. However, this solution is computationally expensive and therefore not ideal for parameter optimisation algorithms. It is also possible to solve the equation analytically, using a standard solution for an inhomogeneous linear differential equation. The analytical solution is difficult to evaluate with respect to numerical limits because, with increasing time, the values of the exponential functions are either large or very low. The solution used in the present study transforms Eq. 1 and 2 to first-order discrete differential equations, which can be solved iteratively. The derivation for Eq. 1 is described below. The resulting Eq. 17 for $b_1(t)$ looks the same as the solution for $b_2(t)$ corresponding to Eq. 2. This is because both equations have identical structure.

The analytical solution for the inhomogeneous linear differential Eq. 1,

$$\frac{\partial^2 \beta}{\partial t^2} + \theta_1 \frac{\partial \beta}{\partial t} + \theta_2 \beta = \alpha(t)$$

is given by

$$\beta(t) = \frac{1}{K} \cdot \left[b_1(t) - b_2(t) \right], \qquad \text{Eq. 16}$$

where we have introduced a constant K and variables $b_1(t)$ and $b_2(t)$:

$$K = \sqrt{\theta_1^2 - 4\theta_2}$$

$$b_1(t) = e^{-t\theta_1} \cdot \int_0^t \alpha(z) e^{\frac{1}{2}z\left(\theta_1 - \sqrt{\theta_1^2 - 4\theta_2}\right)} dz \cdot e^{\frac{1}{2}t\left(\theta_1 + \sqrt{\theta_1^2 - 4\theta_2}\right)}$$

$$b_2(t) = e^{-t\theta_1} \cdot \int_0^t \alpha(z) e^{\frac{1}{2}z\left(\theta_1 + \sqrt{\theta_1^2 - 4\theta_2}\right)} dz \cdot e^{\frac{1}{2}t\left(\theta_1 - \sqrt{\theta_1^2 - 4\theta_2}\right)}$$

If the sampling interval is h, then the expression $b_1(t + h)$ is given by:

$$\begin{aligned} b_1(t+h) &= e^{-\frac{1}{2}(t+h)(\theta_1-K)} \cdot \int_0^{t+h} \alpha(z) e^{\frac{1}{2}z(\theta_1-K)} dz \\ &= e^{-\frac{1}{2}t(\theta_1-K)} e^{-\frac{1}{2}h(\theta_1-K)} \cdot \left[\int_0^{t} \alpha(z) e^{\frac{1}{2}z(\theta_1-K)} dz + \int_t^{t+h} \alpha(z) e^{\frac{1}{2}z(\theta_1-K)} dz \right] \\ &= b_1(t) \cdot e^{-\frac{1}{2}h(\theta_1-K)} + \underbrace{e^{-\frac{1}{2}(t+h)(\theta_1-K)} \cdot e^{\frac{1}{2}(t+h)(\theta_1-K)}}_{1} \cdot \alpha(t+h)h \\ &= b_1(t) \cdot e^{-\frac{1}{2}h(\theta_1-K)} + \alpha(t+h)h \end{aligned} \qquad \text{Eq. 17}$$

Using these equations, the muscle response elicited by a complete spike train can be calculated efficiently without the need to evaluate the analytical solution at each time step, which is computationally expensive. Results were also confirmed by a numerical ordinary differential equation solver in Matlab (Version R14, Mathworks Inc., Natick, Massachusetts).

II. Recursion equation to solve the ODE for the Zajac model

The linear first order Eq. 11 of [3] can be solved by numerical methods applying either a differential equation solver or using a recursion equation. Since the other first order, linear model [4], uses a recursive algorithm to solve the model equation, we converted Eq. 11 to such an equation for fair comparison. As a result, the computational cost is reduced as well. Replacing the differentiation in Eq. 11 by division through a small sampling interval *h* yields:

$$\frac{a(t+h)-a(h)}{h} + \left[\frac{1}{\tau_{act}} (\beta + [1-\beta]u(t+h)) \right] a(t+h) = \frac{1}{\tau_{act}} u(t+h)$$

$$\left[\frac{1}{h} + \frac{1}{\tau_{act}} (\beta + [1-\beta]u(t+h)) \right] a(t+h) = \frac{1}{\tau_{act}} u(t+h) + \frac{a(t)}{h}$$

After rearranging to solve for *a(t + h),*

$$a(t+h) = \left[\frac{1}{h} + \frac{1}{\tau_{act}} (\beta + [1-\beta]u(t+h)) \right]^{-1} \left[\frac{1}{\tau_{act}} u(t+h) + \frac{a(t)}{h} \right] \qquad \text{Eq. 18}$$

References

1. Hatze H (1977) A myocybernetic control model of skeletal muscle . Biol Cybern 25: 103-119.

2. van Zandwijk JP, Bobbert MF, Baan GC, Huijing PA (1996) From twitch to tetanus: performance of excitation dynamics optimized for a twitch in predicting tetanic muscle forces. Biol Cybern 75: 409-417.

3. Zajac FE (1989) Muscle and tendon: properties, models, scaling and application to biomechanics and motor control. Crit Rev Biomed Eng 17: 319-411.

4. Blümel M, Guschlbauer C, Hooper SL, Büschges A (2012) Using individual-muscle specific instead of across-muscle mean data halves muscle simulation error. Biol Cybern 106: 573-585.
